# Cinobufagin-induced DNA damage response activates G_2_/M checkpoint and apoptosis to cause selective cytotoxicity in cancer cells

**DOI:** 10.1186/s12935-021-02150-0

**Published:** 2021-08-23

**Authors:** Jiajing Niu, Jiamei Wang, Qi Zhang, Zhihua Zou, Yushuang Ding

**Affiliations:** 1 Department of Cell Biology and Biophysics, National Engineering Laboratory for AIDS Vaccine, Key Laboratory for Molecular Enzymology and Engineering of the Ministry of Education, School of Life Sciences, Jilin University, Changchun, 130012 China; 2 Department of Radiotherapy, The Second Hospital of Jilin University, Changchun, 130041 China

**Keywords:** Cinobufagin, ROS, Oxidative DNA damage, DNA damage response, Cell cycle arrest, Apoptosis

## Abstract

**Background:**

Processed extracts from toad skin and parotoid gland have long been used to treat various illnesses including cancer in many Asian countries. Recent studies have uncovered a family of bufadienolides as the responsible pharmacological compounds, and the two major molecules, cinobufagin and bufalin, have been shown to possess robust antitumor activity; however, the underlying mechanisms remain poorly understood.

**Methods:**

Intracellular reactive oxygen species (ROS) were measured by DCFH-DA staining and flow cytometry, and DNA damage was analyzed by immunofluorescent staining and the alkaline comet assay. Cytotoxicity was measured by MTT as well as colony formation assays, and cell cycle and apoptosis were analyzed by flow cytometry. In addition, apoptosis was further characterized by TUNEL and mitochondrial membrane potential assays.

**Results:**

Here we showed that sublethal doses of cinobufagin suppressed the viability of many cancer but not noncancerous cell lines. This tumor-selective cytotoxicity was preceded by a rapid, cancer-specific increase in cellular ROS and was significantly reduced by the ROS inhibitor N-acetyl cysteine (NAC), indicating oxidative stress as the primary source of cinobufagin-induced cancer cell toxicity. Sublethal cinobufagin-induced ROS overload resulted in oxidative DNA damage and intense replication stress in cancer cells, leading to strong DNA damage response (DDR) signaling. Subsequent phosphorylation of CDC25C and stabilization of p53 downstream of DDR resulted in activation of the G_2_/M checkpoint followed by induction of apoptosis. These data indicate that cinobufagin suppresses cancer cell viability via DDR-mediated G_2_ arrest and apoptosis.

**Conclusion:**

As elevated oxidative pressure is shared by most cancer cells that renders them sensitive to further oxidative insult, these studies suggest that nontoxic doses of cinobufagin can be used to exploit a cancer vulnerability for induction of cancer-specific cytotoxicity.

**Supplementary Information:**

The online version contains supplementary materials available at 10.1186/s12935-021-02150-0.

## Introduction

Current mainstream anticancer therapies, i.e., chemo- and radiotherapy, target both cancer and normal cells indiscriminately and are often ineffective in the treatment of relapsed tumors. In the past decades, much effort has been devoted to targeting specific genetic alterations or oncogenic signaling pathways [1, 2], however, such approaches have had limited impact on the overall outcome of cancer treatment because of the diversity of cancer genotypes, plasticity of cellular signaling, and intratumor heterogeneity [3–5]. Thus, there is an ongoing and urgent need for novel strategies that target special bioactivities essential to the survival of cancer but not noncancerous cells. With the growing recognition that most cancer cells share rewired metabolic pathways [6–8], targeting cancer metabolism is increasingly viewed as one of the most promising directions to develop broad-spectrum and cancer-selective novel anticancer strategies [9–11].

A marked metabolic feature shared by cells under the influence of oncogenic transformation is elevated generation of reactive oxygen species [12–14]. ROS promote tumorigenesis by causing aberrant cellular signaling and genetic lesions that contribute to irregular cell growth and survival, metastasis, and angiogenesis [15, 16]. However, toxic levels of ROS can damage cellular components to jeopardize cell survival [17, 18]. Many studies have demonstrated that cancer cells are sensitive to additional oxidative insult due to high intrinsic oxidative pressure and limited spare antioxidant capacity [19–22]. Some studies have shown that ROS-generating or antioxidant-suppressing agents can push oxidative pressure to toxic levels selectively in cancer cells [19, 22–25], raising the possibility of exploiting a metabolic vulnerability common to most cancer cells to produce cancer-specific cytotoxicity.

Amphibians secrete a diverse array of chemicals from their skin and special exocrine glands as a defense against microorganisms, predators, and infections [26, 27]. Thus, various forms of extracts from toads, especially members of the Bufonidae family, have been used as traditional medicines worldwide for thousands of years [28]. In China and many other Asian countries, Chansu, a dried preparation of extracts from the skin and parotoid glands of the Asiatic toad (Bufo bufo gargarizans), has long been used in various folk prescriptions to treat pain, infection, inflammation, and cancer [29, 30]. A water-soluble form of the same preparation, called cinobufacini (Huachansu), was developed over two decades ago for injection and was officially approved in China for treatment of various cancers. Both experimental and clinical studies have shown that cinobufacini (Huachansu) possesses significant anticancer efficacy with mild side-effects [31–33], however, due to limited supply of the natural raw drug material and difficulties in controlling drug composition and quality, it is impossible to develop cinobufacini (Huachansu) as a therapeutic drug for cancer treatment.

A family of bufadienolide glycosides (bufadienolides) has been identified as the pharmacological compounds responsible for the antitumor activity of cinobufacini (Huachansu) [34, 35]. Cinobufagin (CBG) and bufalin are the two major bufadienolides in cinobufacini (Huachansu) and other forms of toad extracts, and both have demonstrated potent antitumor efficacy in vitro and in vivo [27, 36]; however, the underlying molecular mechanisms remain poorly understood. Several studies reported that ROS levels were markedly increased in CBG-treated cancer cell lines [37, 38], but the role of ROS in the induction of cancer cell toxicity by CBG has been unclear. In this study, we showed that sublethal doses of CBG were able to induce a rapid increase in cellular ROS levels specifically in cancer cells; CBG-induced ROS overload caused extensive oxidative DNA damage and intense replication stress, and subsequent DDR signaling resulted in activation of the G_2_/M checkpoint and induction of apoptosis to cause cytotoxicity selectively in cancer cells. These studies indicate that CBG-induced oxidative stress is a key factor driving CBG-associated cancer cell toxicity and support further exploration of using CBG to attack a cancer metabolic vulnerability.

## Materials and methods

### Chemicals and cells

Cinobufagin was purchased from Yuanye Biotechnology (Shanghai, China). Stock solution of cinobufagin was prepared in 100% dimethyl sulfoxide (DMSO) (Sigma-Aldrich, St. Louis, MO, USA) and then diluted in complete cell culture medium to make working solutions. The same solutions without cinobufagin were used as vehicle controls.

Human SW480, SW1116 colorectal adenocarcinoma and BEAS-2B bronchial epithelial cell lines were purchased from American Type Culture Collection (ATCC) (Manassas, VA, USA) and the human L-O2 liver and NCM460 colon epithelial cell lines were purchased from KenGen (Nanjing, China). All other cell lines were bought from the Cell Bank of the Chinese Academy of Sciences (Shanghai, China). All cell lines were cultured in Dulbecco’s Modified Eagle’s Medium (DMEM) (Gibco, ThermoFisher Scientific, Shanghai, China) containing 10% fetal bovine serum (FBS) (Gibco, ThermoFisher Scientific, Shanghai, China). The cells were maintained at 37 °C in a humidified incubator with 5% CO2, following instructions from the providers. Cell authenticity was confirmed by short tandem repeats (STR) profiling.

### MTT cell proliferation assay

Cells were seeded in triplicate wells of 96-well plates at 4 × 10^3^ cells per well and treated with cinobufagin (drug concentrations and treatment times were indicated in the figures and figure legends). 20 μl of 5 mg/ml 3-(4,5-dimethylthiazol-2-yl)-2,5-diphenyltetrazolium bromide (MTT) (Sigma-Aldrich) was added to each well, and the plates were kept at 37 °C for another 4 h. After carefully removing the MTT-containing medium, 150 μl of DMSO was added to each well and the plates were kept at 37 °C for 10 min with shaking. Absorbance was read at 595 nm by a BioRad 680 microplate reader (Bio-Rad Laboratories, Hercules, CA, USA). All MTT assays were repeated three times and the data were presented as mean ± standard deviation (SD) of three independent experiments.

### Colony formation assay

Cells seeded in 12-well plates (~ 30% confluence) were treated with the indicated drugs for 5 days, fixed in ice-cold methanol, and then briefly stained with crystal violet solution (0.5% crystal violet in 25% methanol) (Sigma-Aldrich). The violet crystals were dissolved in 70% ethanol, and absorbance at 595 nm was measured.

### Measurement of cellular ROS

Cells were seeded in triplicate wells of 6-well plates at 1 × 10^5^ cells per well and treated with cinobufagin. Cellular ROS were stained by a cell-based ROS assay kit (Beyotime, Shanghai, China) following manufacturer’s instructions. Briefly, cells were washed with PBS, and incubated with 10 μM (DCFH-DA) for 30 min at 37 °C in the dark. After washing three times in PBS, photos were taken immediately using an Olympus fluorescent microscope. The cells were then collected through trypsinization and analyzed on the BD FACS-Calibur flow cytometer (BD Biosciences, San Jose, CA, USA). Cellular ROS levels were expressed as the average 2’,7’-dichlorofluorescein fluorescence intensity. Results were mean ± SD of three independent experiments.

### Immunofluorescent staining

Cells were seeded on round coverslips in 24-well plates and treated with cinobufagin. After washing in PBS, cells were fixed in ice-cold 4% paraformaldehyde (PFA) for 30 min and washed three times with phosphate buffer saline (PBS).

For immunostaining of 8-oxoguanine (8-oxoGua), fixed cells were incubated in Alexa 488-conjugated avidin (Rockland Immunochemicals, Limerick, PA, USA) (0.5 mg/ml) for 1 h at room temperature, washed three times in PBS, and the coverslips with stained cells were sealed on glass slides in the VECTASHIELD Mounting Medium with DAPI (Vector Laboratories, Burlingame, CA, USA). Images were acquired using a Zeiss LCM 510 confocal microscope, signal intensity was quantified using the ImageJ software. At least 50 cells per sample were measured.

For immunofluorescent staining of 53BP1 or γH2AX, the cells were incubated sequentially in blocking buffer (3% fetal bovine serum, 0.1% Triton X-100 in PBS), rabbit anti-53BP1 (Bethyl Laboratories, Montgomery, TX, USA) or rabbit-anti-γH2AX-pS139 (Abcam, Cambridge, UK) primary antibody, and Cy3-conjugated goat anti-rabbit secondary antibody (Jackson ImmunoResearch Laboratories, West Grove, PA, USA), each for 1 h at room temperature. Cells were then washed three times in PBS, and the coverslips with stained cells were sealed on glass slides in the VECTASHIELD Mounting Medium with DAPI (Vector Laboratories). Images were acquired using a Zeiss LCM 510 confocal microscope. Foci were quantified using the ImageJ software, more than 100 cells per sample were analyzed.

### Comet assay

Measurement of DNA strand breaks in individual cells by the alkaline comet assay (single cell gel electrophoresis assay) was performed according to the instructions included in the OxiSelect Comet Assay kit (Cell Biolabs, San Diego, CA, USA). Cells grown in 6-well plates were treated with cinobufagin and collected through trypsinization. Cell pellets were resuspended in 1.2% low-melting point agarose maintained at 37 °C at 10 × 10^5^ cells/ml, which were then layered on a frosted slide from the OxiSelect Comet Assay kit. The slides were stored at 4 °C overnight in pre-cooled lysis buffer (100 mM EDTA, 2.5 M NaCl, 10 mM Tris–HCl, 1% Triton X-100 and 10% DMSO, pH 10.0). After washing twice with an enzyme buffer (40 mM HEPES, 0.1 M KCl, 0.5 mM EDTA and 0.2 mg/ml BSA, pH 8.0), the slides were incubated in the enzyme buffer with or without 1.0 μg/ml OGG1 (ProSpec, Rehovot, Israel) for 45 min at 37 °C, washed briefly in the enzyme buffer, and denatured in pre-chilled alkali buffer (300 mM NaOH, 1 mM EDTA) in a horizontal electrophoresis chamber (Bio-Rad Laboratories) for 30 min. Electrophoresis was then proceeded at 20 V and 300 mA in the same buffer for 30 min. After incubation in cold neutralizing buffer (250 mM Tris–HCl, pH 7.5) for 30 min, slides were immersed in cold 70% ethanol for 5 min and allowed to air dry. At the end, cells were stained with Vista Green DNA dye provided by the kit at room temperature for 15 min. Images were acquired with an Olympus fluorescent microscope and quantified using the Comet Assay IV software (Perceptive Instruments, Edmunds, UK). The tail moment was defined as the product of the tail length and the fraction of total DNA in the tail, at least 50 cells per sample were analyzed.

### Flow cytometric analysis of cell cycle and apoptosis

Cells were seeded in triplicate wells of 6-well plates at 1 × 10^5^ cells per well, treated with cinobufagin, and collected through trypsinization. The cell pellets were washed twice in PBS before the following analyses. All experiments were performed three times and the results were presented as mean ± SD.

For cell cycle analysis, cell pellets were fixed in 70% ice-cold ethanol at − 20 °C for 1 h. After washing in PBS twice, cells were stained with working solutions from the Cell Cycle Detection kit (BestBio, Shanghai, China). Samples were loaded onto and analyzed by the MoFlo XDP Cell Sorter (Beckman Coulter, Indianapolis, IN, USA). Data were processed with the FlowJo software (FlowJo, Ashland, OR, USA).

For the analysis of apoptosis, cells were resuspended in a binding buffer from the Annexin V-FITC Apoptosis Detection kit (BestBio) according to the instructions provided by the manufacturer. The cells were loaded onto and analyzed by the MoFlo XDP Cell Sorter (Beckman Coulter) and data were processed with the CytExpert software (Beckman Coulter).

### TUNEL assay

Cells were seeded on round coverslips in 24-well plates and treated with cinobufagin. After washing in PBS, cells were fixed in ice-cold 4% paraformaldehyde (PFA) for 30 min and washed three times with PBS. Fixed cells were incubated in 0.1% Triton X-100 in PBS for 2 min on ice, washed twice with PBS, and then incubated in a working solution from the One-Step TUNEL Apoptosis Assay kit (Beyotime) for 60 min at 37 °C. After washing three times in PBS, the coverslips with stained cells were sealed on glass slides in the VECTASHIELD Mounting Medium with DAPI (Vector Laboratories). Photos were captured using a Zeiss LCM 510 confocal microscope.

### Measurement of MMP

Cells were seeded in triplicate wells of 6-well plates at 1 × 10^5^ cells per well and treated with cinobufagin. Mitochondrial membrane potential (MMP) was measured using a commercial Mitochondrial Membrane Potential Assay kit with JC-1 (Beyotime) following manufacturer’s instructions. Images were taken using an Olympus fluorescent microscope.

### Western blot

Cells grown in 6-well plates were scraped off the plates in 100 μl of radioimmunoprecipitation buffer (150 mM NaCl, 1.0% IGEPAL CA-630, 0.5% sodium deoxycholate, 0.1% sodium dodecyl sulfate, and 50 mM Tris, pH 8.0) (Sigma-Aldrich) with 1 mM phenylmethane sulfonylfluoride (Sigma-Aldrich). Samples were centrifuged at 4 °C for 20 min at 12,000 × g and protein concentrations were determined by a BCA Protein Assay kit (Dingguo, Changchun, China). Proteins were denatured at 95 °C for 10 min, separated on a 12% SDS-PAGE gel, and transferred to PVDF membranes. The membranes were blocked in 5% (w/v) non-fat milk in TBST (10 mM Tris, 100 mM NaCl, 0.1% Tween 20, pH 7.5) for 1 h at room temperature, and then incubated sequentially in primary and secondary antibodies each for 1 h at room temperature. Signals were developed using a Tanon-5200 chemiluminescence image analysis system (Tanon, Shanghai, China). All experiments were performed three times.

Primary antibodies included γH2AX-pS139 polyclonal antibody (ab11174) (Abcam, Cambridge, UK); Chk1-pS317 (12302S) and Chk2-pT68 (2661S) (Cell Signaling, Danvers, MA, USA); Chk1 (bs-1681R), GAPDH (bs-2188R), CDC25C (bs-10579R), activated caspase 3 (bsm-33199 M), caspase 3 (bsm-52289R), CDC25C-pS216 (bs-3096R), cyclin B (bs-6656R), p53-pS15 (bs-3702R) and p53 (bs-2092R) (Bioss, Beijing, China); CDC25A (abs131784) and Chk2 (abs131635) (Absin Bioscience, Shanghai, China); 53BP1 (A300-272A) (Bethyl, Montgomery, TX, USA); Phospho-CDK1 (AF5761) (Beyotime, Shanghai, China); RPA2 (NBP1-23017) (NOVUS, Shanghai, China). Secondary antibodies were goat anti-mouse-Alexa 488 (115-545-003) and goat anti-rabbit-Cy3 (111-165-003) (Jackson ImmunoResearch, West Grove, PA, USA); goat anti-mouse-HRP (bs-40296G-HRP) and goat anti-rabbit-HRP (bs-40295G-HRP) (Bioss).

### Mouse xenograft study

Animal studies were performed in compliance with animal protocols approved by the Institutional Animal Care and Use Committee of Jilin University. SW1116 cells (2 × 10^6^) were inoculated subcutaneously into the flank of 6-week-old athymic BALB/c nude mice (Charles River, Boston, MA, United States). The animals were randomly placed in control and treatment groups (7 mice per group). When tumors reached about 100 mm^3^, the mice were treated once daily with 2, 5 or 10 mg/kg cinobufagin oral gavage (p.o.) for 3 weeks. Tumor volume was measured every day, and tumor weight was measured at the end of treatment. 5 μm-thick paraffin sections of heart, liver, and kidney tissues were stained with hematoxylin–eosin solutions.

### Statistical analysis

Statistical analyses were carried out in GraphPad Prism 7 (GraphPad Software, San Diego, CA, USA). Comparisons between two groups were performed by unpaired two‑tailed Student's *t*‑test, and *p* < 0.05 was considered statistically significant.

## Results

### Sublethal cinobufagin increases ROS levels specifically in cancer cells

**Fig. 1:**
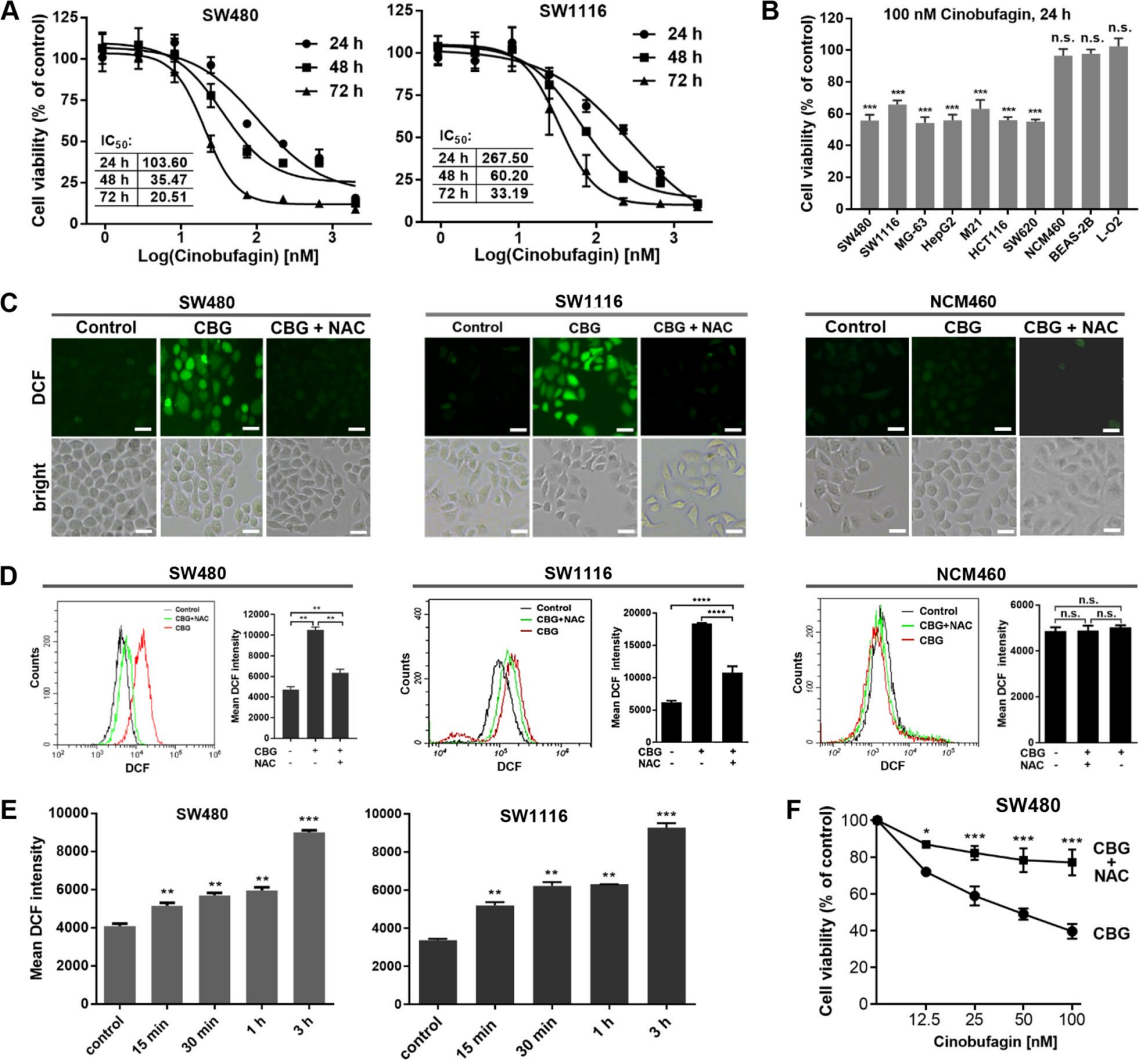
Sublethal cinobufagin increases ROS levels specifically in cancer cells. **A** Measurement of IC_50_ by MTT. SW480 and SW1116 cancer cells were treated by 0.9, 2.7, 8.2, 24.7, 74.0, 222.2, 666.7 or 2000.0 nM CBG for 24, 48 or 72 h. IC_50_ values were derived in the Prism 7 software. **B** Evaluation of cytotoxicity by MTT. Cells were treated by 100 nM CBG for 24 h. Cell viability was shown as the percentage of vehicle control of the corresponding cell line. Viability of the cancer but not noncancerous cell lines was reduced by 40–50%. **C** Representative images of cells stained by DCFH-DA. Cells were treated by 100 nM CBG for 3 h. Oxidization of DCFH-DA-derived, membrane-impermeable DCFH generated highly fluorescent 2’,7’-dichlorofluorescein (DCF). Intensity of DCF fluorescence served as a measure of cellular ROS. A prominent increase in ROS levels was induced in SW480 and SW1116 but not NCM460 cells, and NAC blocked the ROS increase in the cancer cells (scale bar: 25 μm). **D**, **E** Measurement of ROS levels by flow cytometry. Treatment by 100 nM CBG for 3 h caused a pronounced increase in ROS levels in SW480 and SW1116 but not NCM460 cells, NAC blocked the ROS increase in SW480 and SW1116 cells (**D**); significant increase in ROS levels in the cancer cells was evident 15 min after treatment by 100 nM CBG (**E**). **F** MTT assay. SW480 cancer cells were treated with 12.5, 25, 50 or 100 nM CBG, with or without NAC, for 24 h. NAC significantly reduced the cytotoxicity induced by CBG. *n.s.:* not significant, *: *p* < 0.05, **: *p* < 0.01, ***: *p* < 0.001 vs vehicle control or NAC-treated group (n = 3)

To evaluate the in vitro anticancer potency of cinobufagin and determine the dose range for subsequent experiments, we studied the IC_50_ values of CBG against the human colorectal adenocarcinoma cell lines SW480 and SW1116. After treatment with CBG for 24, 48 or 72 h, the IC_50_ values were 103.60, 35.47 or 20.51 nM in SW480 cells and 267.50, 60.20 or 33.19 nM in SW1116 cells (Fig. 1A), all were in the low nanomolar range, indicating that CBG was potently cytotoxic in these cancer cells.

Similar to the SW480 and SW1116 colorectal cancer cells, the viability of some diverse human cancer cell lines, including the osteosarcoma MG-63, hepatocellular carcinoma HepG2, melanoma M21, and two other colorectal carcinoma cell lines HCT116 and SW620, was also reduced by 40–50% after 24 h of treatment with 100 nM CBG, a dose close to the IC_50_ value of 24-h treatment in SW480 cancer cells (Fig. 1B). Similar results were also showed by a 5-day colony formation assay with the pancreatic epithelioid carcinoma PANC-1, cervical squamous cell carcinoma SiHa, hepatocellular carcinoma HepG2, lung epithelial carcinoma A549 and adenocarcinoma HCC827, and the colorectal adenocarcinoma SW480 and SW1116 cells (Additional file 1: Figure S1A). Together, these results demonstrated a similar degree of CBG-induced cytotoxicity in a broad range of cancer cell types. In stark contrast, the viability of three noncancerous human cell lines, including the NCM460 colon and BEAS-2B lung epithelial cell lines, and the L-O2 hepatocyte cell line, was almost not affected by the same treatment (Fig. 1B, Additional file 1: Figure S1A), revealing an interesting difference between cancer and noncancerous cells towards CBG’s cytotoxicity-inducing activity.

Oncogenic transformation is associated with increased generation of ROS and agents that promote generation of additional ROS or weaken the antioxidant systems may push oxidative pressure to toxic levels selectively in cancer cells, resulting in cancer-specific oxidative toxicity. CBG has been shown to markedly increase ROS levels in cancer cells. To understand the mechanisms underlying the differential cytotoxicity of CBG in cancer and noncancerous cells, we measured cellular ROS levels by the oxidant-sensing probe 2’,7’-dichlorodihydrofluorescein diacetate (DCFH-DA). Remarkably, a prominent increase in ROS levels was revealed in the SW480 and SW1116 colorectal cancer cells after 3 h of treatment by 100 nM CBG (Fig. 1C, D); in contrast, similar treatments caused no change in ROS levels in the noncancerous NCM460 colon epithelial cells (Fig. 1C, D). Similarly, a marked increase in ROS levels was induced by the same treatment in the lung epithelial carcinoma A549 and HCC827 and the hepatocarcinoma HepG2 cells but not in the noncancerous BEAS-2B lung epithelial cells and the L-O2 hepatocytes (Additional file 1: Figure S1B–C). Significant ROS increase in the cancer cells was evident as early as 15 min after 100 nM CBG treatment (Fig. 1E), and the ROS inhibitor N-acetyl cysteine effectively blocked the ROS increase in the cancer cells (Fig. 1C, D, Additional file 1: Figure S1B, C). Interestingly, NAC also significantly reduced the cytotoxicity of CBG in cancer cells (Fig. 1F), correlating CBG-induced cytotoxicity with ROS elevation. Thus, a rapid increase in ROS levels was induced specifically in cancer cells by a sublethal dose of CBG, and ROS elevation resulted in cytotoxicity selectively in cancer cells.

### Sublethal cinobufagin-induced ROS overload leads to oxidative DNA damage

Elevated ROS can cause oxidative DNA damage that may lead to cell cycle arrest, premature cellular senescence, or programmed cell death if the damage results in intense DNA damage response signaling. To investigate if activation of DDR was responsible for CBG-induced suppression of cancer cell viability, we first checked whether oxidative DNA damage was resulted from CBG-induced ROS elevation.

**Fig. 2:**
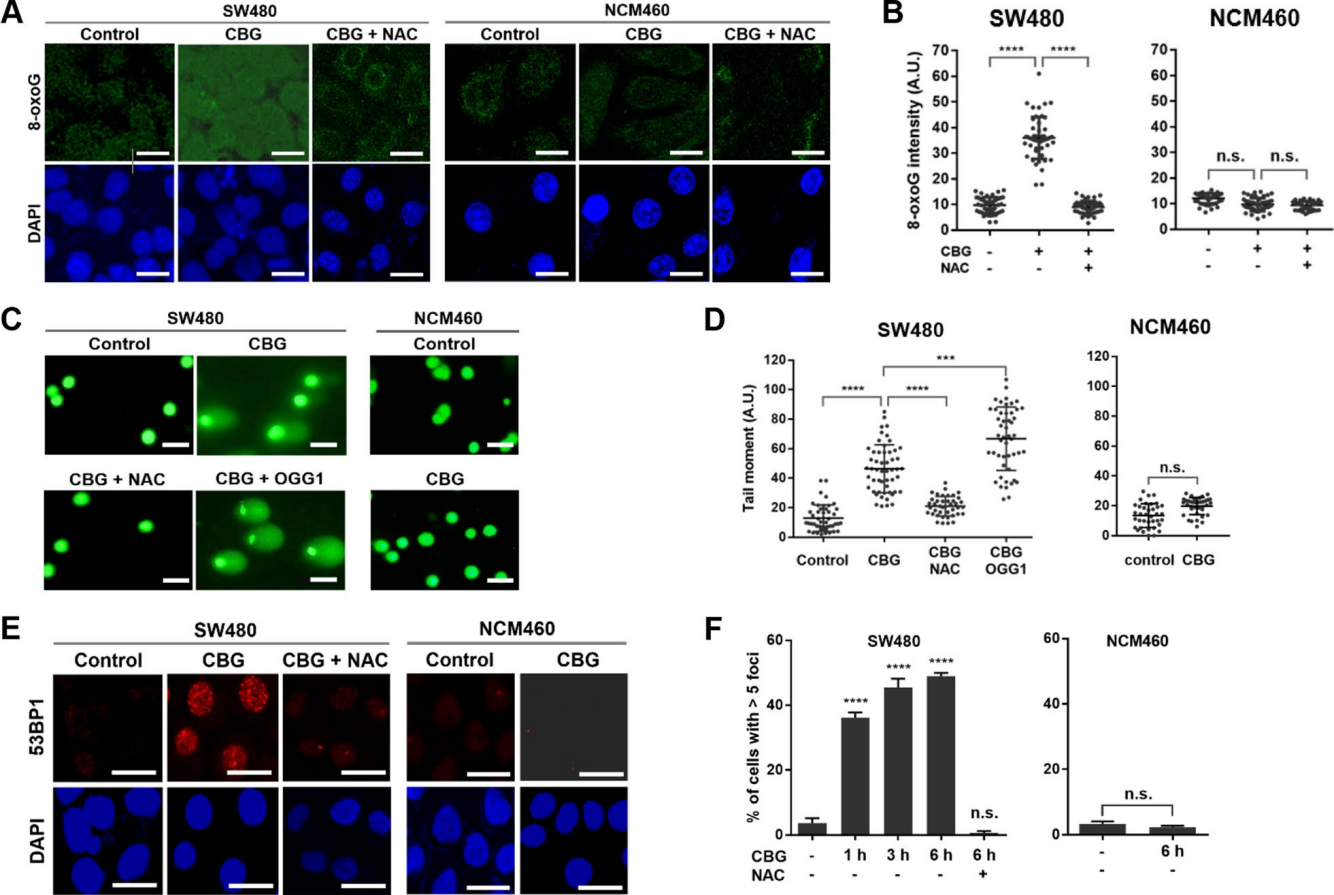
Cinobufagin-induced ROS overload results in oxidative DNA damage. **A** Representative images of 8-oxoGua immunostaining (scale bar: 10 μm) and **B** Quantification of 8-oxoGua intensity in single cells. Cells were treated by 100 nM CBG for 3 h. Nuclear 8-oxoGua intensity was quantified by ImageJ, at least 50 cells per sample were analyzed. **C** Representative images of alkaline comet assay (scale bar: 25 μm) and **D** Quantification of tail moment in single cells. Cells were treated by 100 nM CBG for 3 h. Tail moment was defined as the product of tail length and fraction of total DNA in the tail and was quantified by the Comet Assay IV software. At least 50 cells per sample were analyzed. **E** Representative images of 53BP1 immunostaining (scale bar: 25 μm) and **F** Quantification of 53BP1-positive cells. Cells were treated by 100 nM CBG for 3 h (**E**) or for the indicated times (**F**). At least 5000 cells per treatment group were analyzed. *n.s.*: not significant, ***: *p* < 0.001, ****: *p* < 0.0001 vs vehicle control or NAC-treated group (*n* = 3)

One of the most common targets of ROS is the nucleobase guanine in both nucleic acid macromolecules (DNA and RNA) and the free nucleotides dGTP and GTP. Oxidation of guanine generates 8-oxoguanine (8-oxoGua) which can be revealed by labeled avidin [39]. Immunofluorescent staining using Alexa 488-conjugated avidin showed that treatment with 100 nM CBG for 3 h markedly increased nuclear 8-oxoGua levels in the SW480 cancer but not in the NCM460 noncancerous colon epithelial cells (Fig. 2A, B). Similarly, a significant increase in 8-oxoGua levels was induced in the A549 lung epithelial and HepG2 hepatocellular carcinoma but not in the noncancerous BEAS-2B lung epithelial cells and the L-O2 hepatocytes (Additional file 2: Figure S2A), demonstrating the presence of CBG-induced, cancer-specific oxidative DNA damage. NAC effectively blocked production of 8-oxoGua in the cancer cells (Fig. 2A, B, Additional file 2: Figure S2A), correlating 8-oxoG generation with CBG-induced ROS overload.

Another major form of oxidative DNA damage is single-strand DNA breaks (SSBs) produced either directly through ROS-mediated oxidization or as intermediates of base excision repair (BER) of oxidized nucleobases. Double-strand DNA breaks (DSBs) may be generated when DNA replication forks collide with SSBs or DNA repair complexes during DNA replication. The alkaline comet assay provides a measure of total DNA strand breaks in single cells because alkaline treatment converts all SSBs into DSBs. The results of alkaline comet assay showed that treatment by 100 nM CBG for 3 h greatly increased the number of total DNA strand breaks in the SW480, A549 and HepG2 cancer but not in the NCM460, BEAS-2B and L-O2 noncancerous cells, and NAC effectively blocked the generation of DNA breaks (Fig. 2C, D, Additional file 2: Figure S2B).

OGG1 is the glycosylase that removes 8-oxoGua and cut the DNA strand to generate an SSB during BER. Pre-incubation with OGG1 significantly increased the number of DNA breaks revealed by alkaline comet assay in the cancer cells (Fig. 2C, D, Additional file 2: Figure S2B), indicating the presence of a large number of 8-oxoGua in the cancer DNA.

To check if DSBs were produced in CBG-treated cancer cells, we stained 53BP1, a protein that concentrates at sites of DSB to yield 53BP1 staining foci, therefore allowing direct visualization and measurement of DSBs. Immunofluorescent staining showed that 3 h of treatment by 100 nM CBG markedly increased the number of SW480, A549 and HepG2 cancer cells with strongly stained nuclear 53BP1 foci (Fig. 2E, Additional file 2: Figure S2C), while no change in 53BP1 staining signal was evident in similarly treated NCM460, BEAS-2B and L-O2 noncancerous cells (Fig. 2E, Additional file 2: Figure S2C). Increase in the number of 53BP1-positive cancer cells became significant 1 h after treatment by 100 nM CBG and continued in a time-dependent manner (Fig. 2F). NAC effectively blocked generation of 53BP1 foci (Fig. 2E-F, Additional file 2: Figure S2C). These results showed that some of the DNA strand breaks detected by the alkaline comet assay were DSBs and confirmed the presence of DSBs, the most toxic form of DNA damage, in CBG-treated cancer cells.

Together, these data demonstrated that sublethal CBG-induced ROS elevation immediately resulted in extensive oxidative DNA damage, including DSBs, specifically in cancer cells.

### Replication stress and DDR are resulted from cinobufagin-induced DNA damage

Acute generation of extensive DNA damage may result in collision between moving DNA replication forks and damaged DNA or DNA repair complexes, leading to generation of DSBs and replication stress, both of which may activate the ATM-Chk2 or ATR-Chk1 DDR signaling pathway.

**Fig. 3:**
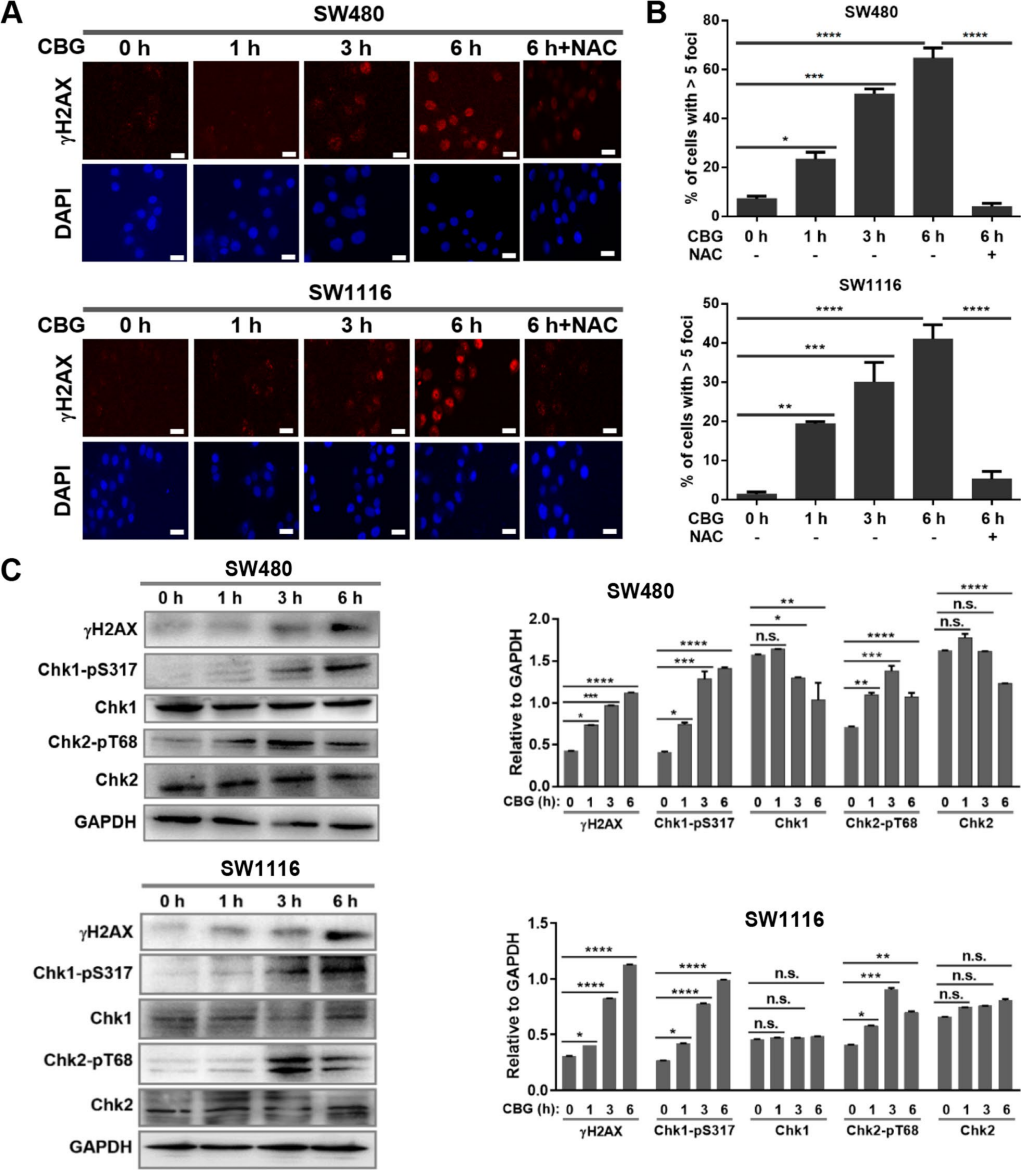
Cinobufagin-induced oxidative DNA damage leads to replication stress and activation of DNA damage response. **A** Representative images of γH2AX immunostaining (scale bar: 10 μm) and **B** Quantification of γH2AX-positive cells. Cells were treated by 100 nM CBG for the indicated times. At least 5000 cells per treatment group were analyzed. Treatment by 100 nM CBG induced a rapid, time-dependent increase in both the intensity of γH2AX staining signal and the number of γH2AX-positive cells. **C** Western blot. Cells were treated by 100 nM CBG for the indicated times. A time-dependent, rapid increase in levels of γH2AX and phosphorylated Chk1 and Chk2 was induced by CBG treatment. *n.s.:* not significant, *: *p* < 0.05, **: *p* < 0.01, ***: *p* < 0.001, ****: *p* < 0.0001 vs vehicle control or NAC-treated group (*n* = 3)

Indeed, in the SW480 and SW1116 colorectal cancer as well as the A549 lung and HepG2 hepatocellular carcinoma cells, treatment by 100 nM CBG produced a rapid and progressive increase in the number of cells with strong pan-nuclear γH2AX staining, which was not seen in the noncancerous NCM460, BEAS-2B and L-O2 cells (Fig. 3A, B, Additional file 3: Figure S3A). A marked increase in the levels of γH2AX was also revealed by Western blot analyses in the cancer but not noncancerous cells (Fig. 3C, Additional file 3: Figure S3B), indicating the presence of intense replication stress. Consistently, a time-dependent increase in phospho-RPA32 (RPA32-pS4, S8) levels was demonstrated by Western blot in SW480 and SW1116 colorectal cancer cells (Additional file 3: Figure S3C). Generation of γH2AX-positive cells and the increase in γH2AX levels in the cancer cells were effectively blocked by NAC (Fig. 3A, B, Additional file 3: Figure S3A–B), correlating CBG-induced replication stress with induction of oxidative DNA damage.

Similar to induction of replication stress, Western blot analyses also showed a time-dependent, significant increase in levels of phosphorylated Chk1 and Chk2, demonstrating strong activation of both ATR-Chk1 and ATM-Chk2 DDR signaling pathways specifically in the cancer but not noncancerous cells (Fig. 3C, Additional file 3: Figure S3B). Again, increase in phosphorylation of Chk1 and Chk2 in the cancer cells was reversed by NAC (Additional file 3: Figure S3B). The induction of increase in the levels of phosphorylated γH2AX, Chk1 and Chk2 in the cancer cells by CBG was similar to that induced by a positive control H_2_O_2_ (Additional file 3: Figure S3D).

Interestingly, DSBs, represented by 53BP1 foci, became markedly increased 1 h after CBG-treatment (Fig. 2F), while phosphorylation of Chk2, which is stimulated mainly by DSB, peaked after 3 h of CBG treatment (Fig. 3C), and phosphorylation of Chk1, which is stimulated primarily by replication stress, became significantly increased after 6 h of CBG treatment (Fig. 3C). Taken together, these results showed that CBG-induced oxidative DNA damage resulted in generation of DSBs and intense replication stress, which subsequently caused the activation of the ATM-Chk2 and ATR-Chk1 DDR signaling pathways, respectively.

### DDR signaling activates the G_2_/M cell cycle checkpoint

DDR signaling can cause cell cycle arrest in G_1_, S or G_2_ phase via mechanisms including phosphorylation-mediated inactivation of the phosphatase CDC25 and/or upregulation of the p53-p21^Cip1/Waf1^ axis; p53 can also upregulate pro-apoptotic proteins to induce apoptosis. Cell cycle arrest and apoptosis will both result in suppression of cell viability.

**Fig. 4:**
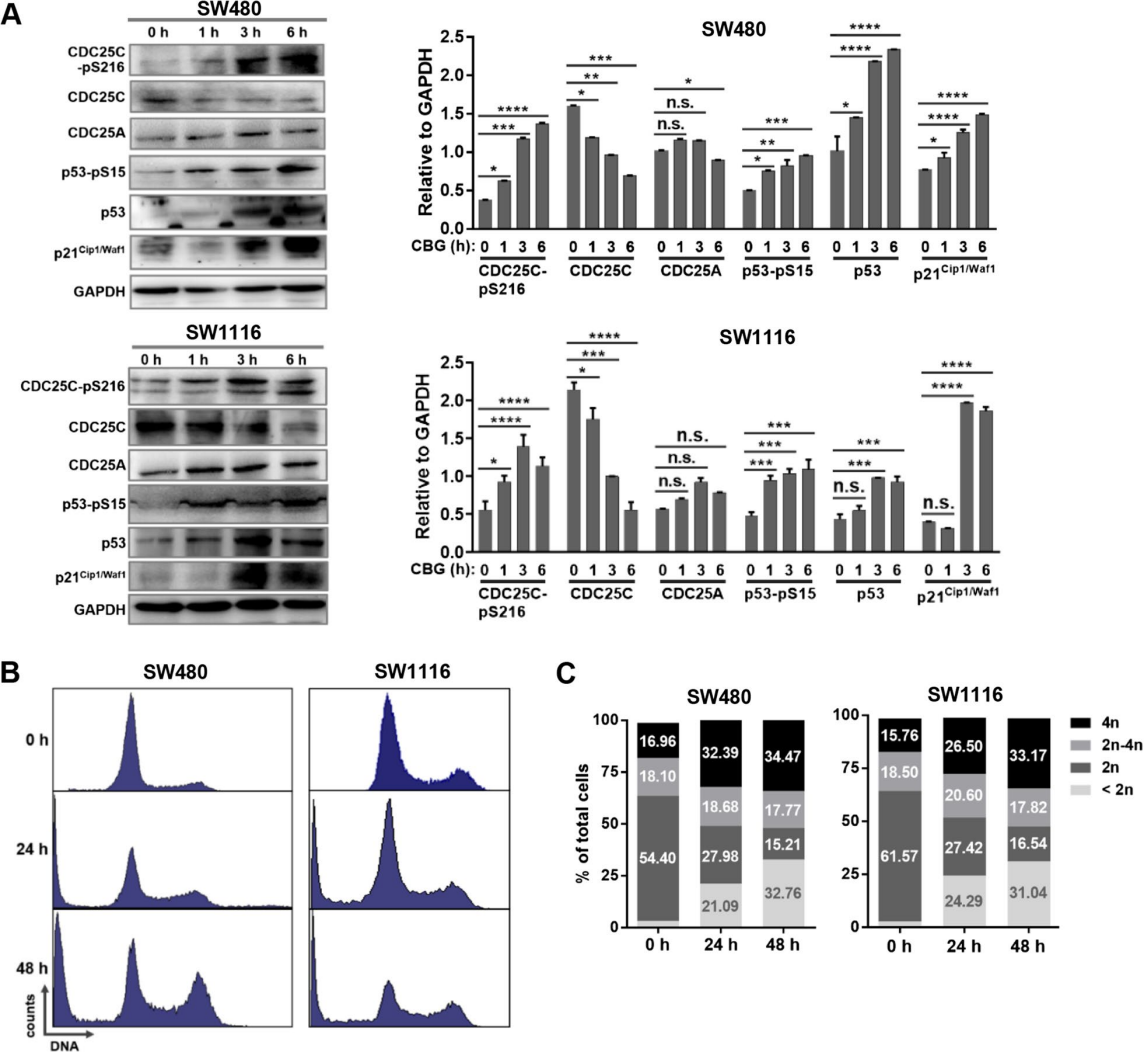
DDR signaling activates the G_2_/M checkpoint to induce G_2_ arrest. **A** Western blot. Cells were treated by 100 nM CBG for the indicated times. A time-dependent, rapid increase in phosphorylation of CDC25C and p53, as well as total protein levels of p53 and p21^Cip1/Waf1^, was induced. **B**, **C** Analysis of cell cycle by flow cytometry. Cells were treated by 100 nM CBG for 24 or 48 h. Treatment by 100 nM CBG caused a progressive accumulation of cells in the 4n group, a rapid decrease in the size of the 2n population and no significant change in the size of the 2n-4n population. The subG_1_ population (cells with < 2n DNA) increased continuously. Some cells with > 4n DNA were generated during the first 24 h but disappeared after 48 h of treatment (**B**), they were excluded from quantitative measurements (**C**). *n.s.:* not significant, *: *p* < 0.05, **: *p* < 0.01, ***: *p* < 0.001, ****: *p* < 0.0001 vs vehicle control (*n* = 3)

In the SW480 and SW1116 cancer cells, Western blot analyses showed that treatment by 100 nM CBG induced a significant, progressive decrease in the levels of cyclin B and conspicuous accumulation of CDK1-pT15 (Additional file 3: Figure S3C), indicating activation of the G_2_/M cell cycle checkpoint. Consistently, treatment by 100 nM CBG induced a time-dependent, prominent increase in phosphorylation of CDC25C, the key driver of G_2_/M transition, and a corresponding decrease in the total protein levels of CDC25C (Fig. 4A), demonstrating that CDC25C was rapidly inactivated by DDR signaling to block G_2_/M transition, i.e., to activate the G_2_/M cell cycle checkpoint. The levels of both phosphorylated and total p53 proteins, as well as p21^Cip1/Waf1^, were similarly increased in the CBG-treated SW480 and SW1116 cancer cells, indicating activation of the p53-p21^Cip1/Waf1^ axis (Fig. 4A), which could promote activation of the G_1_, S or G_2_/M checkpoints.

Consistent with the results of Western blot indicating activation of the G_2_/M checkpoint, flow cytometry analyses showed that treatment by 100 nM CBG caused a rapid and progressive accumulation of SW480 and SW1116 cancer cells in the 4n group and a fast decrease in the size of the 2n population (Fig. 4B, C), confirming the induction of G_2_ arrest. The size of the 2n-4n population showed nearly no change (Fig. 4B, C), suggesting little or no induction of cell cycle arrest in S phase, which was consistent with the constant protein levels of CDC25A (Fig. 4A).

### Apoptosis is induced after G_2_ arrest

**Fig. 5:**
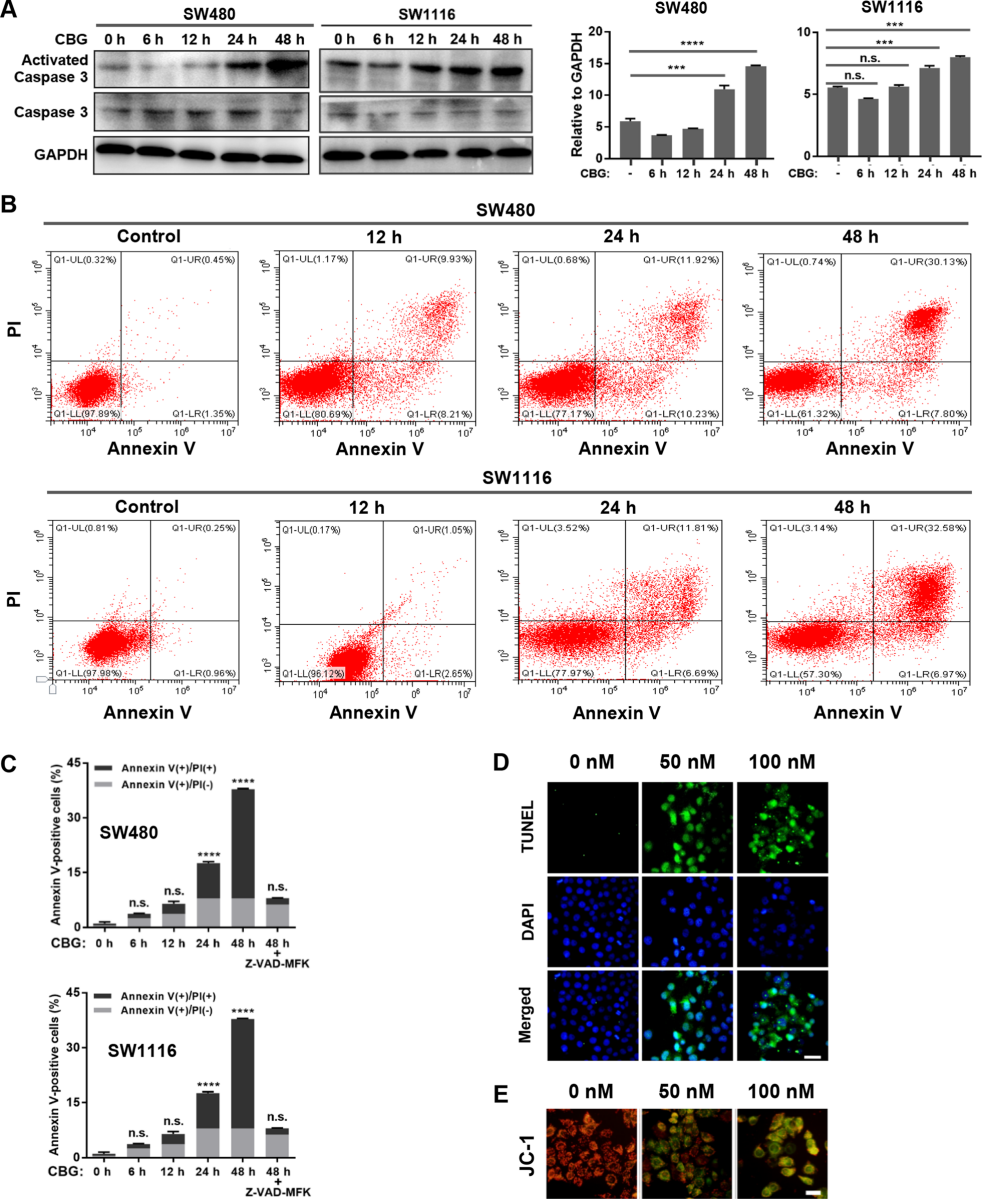
Apoptosis is induced after G_2_ arrest. **A** Western blot. Cells were treated by 100 nM CBG for the indicated times. Levels of activated caspase 3 were markedly increased after treatment by 100 nM CBG for 24 h. **B**, **C** Flow cytometry analysis of apoptosis. Cells were treated by 100 nM CBG for the indicated times. The number of Annexin V-positive cells increased dramatically after 24 h of treatment by 100 nM CBG. The pan-caspase inhibitor Z-VAD-FMK blocked the increase in Annexin V-positive cells. **D** TUNEL assay. SW480 cancer cells were treated by the indicated doses of CBG for 24 h. Apoptotic cells were labeled by FITC-conjugated dUTP. CBG treatment dose-dependently increased the degree of apoptosis (scale bar: 50 μm). **E** Measurement of mitochondrial membrane potential. SW480 cancer cells were treated by the indicated doses of CBG for 24 h and stained with JC-1. The mitochondrial membrane potential probe JC-1 forms aggregate in the mitochondria of healthy cells and emits red fluorescence. Loss of mitochondrial membrane potential causes JC-1 to disperse into monomers and emits green fluorescence. CBG treatment dose-dependently increased the intensity of green fluorescence, while the intensity of red fluorescence decreased (scale bar: 25 μm). *n.s.: * not significant, ***: *p* < 0.001, ****: *p* < 0.0001 vs vehicle control (*n* = 3)

Cell cycle analyses by flow cytometry showed that treatment by 100 nM CBG resulted in a fast and continuous increase in the size of the subG_1_ population (cells with < 2n DNA), which reached ~ 33% after treatment by 100 nM CBG for 48 h (Fig. 4B, C), indicating a time-dependent, steady increase of cell death. To evaluate the modes of cell death, we examined the levels of cleaved caspase 3 by Western blot. The results showed that the amount of activated caspase 3 was markedly increased after 24 h of treatment by 100 nM CBG and reached much higher levels after 48 h of treatment (Fig. 5A), suggesting significant induction of caspase-dependent apoptosis. CDC25C was intensely phosphorylated within hours of CBG treatment to implement G_2_ arrest (Fig. 4A), while caspase 3 became significantly activated after 24 h of CBG treatment (Fig. 5A), suggesting apoptosis was induced after G_2_ arrest, likely as a consequence of extended G_2_ arrest and DDR signaling due to persistent stress and/or unresolvable damage.

**Fig. 6:**
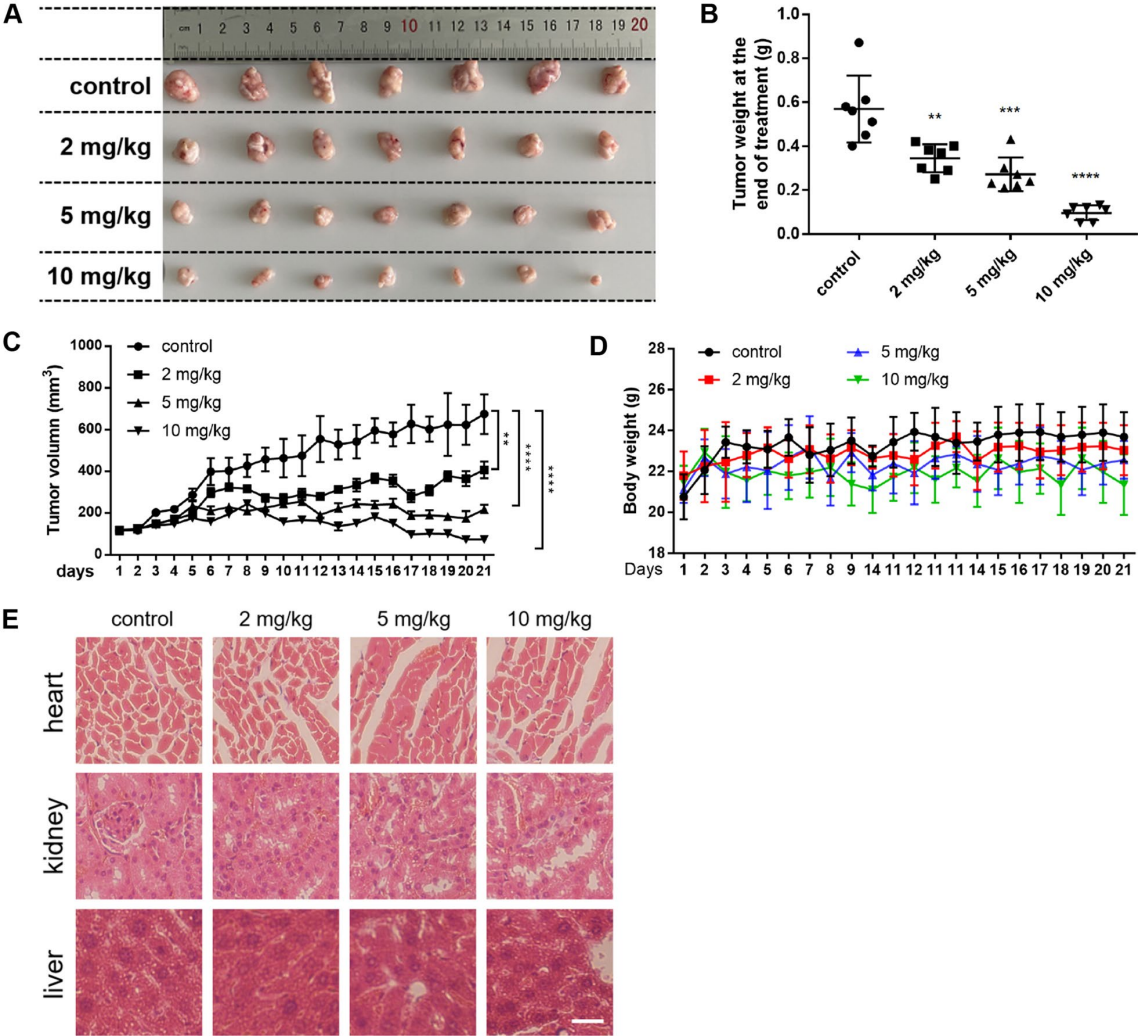
Cinobufagin induces regression of tumor xenografts in vivo. **A** A photograph of tumor mass dissected out at the time of study termination. **B** Tumor weight measured at the end of the study. **C** Tumor volume-time curve. **D** Body weight-time curve. (**E**) Hematoxylin-eosin-stained sections of liver, heart, and kidney tissues (magnification: ×400). *n.s.:* not significant, **: *p* < 0.01, ***: *p* < 0.001, ****: *p* < 0.0001 vs. vehicle control

Consistent with the increase in caspase 3 activation, flow cytometry analyses showed that the number of Annexin V-positive SW480 and SW1116 cancer cells was significantly increased by CBG treatment in a time-dependent manner, reaching ~ 38% after treatment by 100 nM CBG for 48 h (Fig. 5B, C). The pan-caspase inhibitor Z-VAD-FMK blocked the increase of Annexin V-positive cells (Fig. 5C), confirming the induction of caspase-dependent apoptosis by a sublethal dose of CBG.

Moreover, labeling of apoptotic cells by the TUNEL assay revealed that sublethal doses of CBG induced a marked, dose-dependent increase in TUNEL-positive SW480 cancer cells (Fig. 5D), and measurement of MMP by the JC-1 probe demonstrated a dose-dependent induction of MMP dissipation (Fig. 5E). These results further confirmed the activation of the mitochondrial apoptosis pathway by sublethal doses of CBG in the cancer cells, following DNA damage-induced DDR signaling.

### Cinobufagin induces regression of tumor xenografts in vivo

To assess the clinical potential of CBG, we evaluated the effects of treatment with CBG on tumor xenografts. Nude mice bearing SW1116 tumor xenografts were treated with either PBS (vehicle control) or CBG (dosed once daily by oral gavage, at 2, 5 or 10 mg/kg), for 20 days. The results showed that all three doses of CBG significantly inhibited the growth of the tumor, with the 10 mg/kg dose induced substantial regression of established tumors (Fig. 6A–C), demonstrating in vivo efficacy for CBG. No significant difference in body weight was observed between control and the drug-treated groups (Fig. 6D), and microscopic examination of hematoxylin–eosin stained tissue sections of liver, heart, and kidney showed normal histological morphology and structure for all groups, suggesting that the drug was well tolerated.

## Discussion

Chansu is a dried preparation of extracts from the skin and parotoid glands of the Asiatic toad and has long been used in various folk formulations to treat a number of illnesses including pain, infection, inflammation and cancer in many Asian countries [27, 29, 30]. Cinobufacini (Huachansu) is the soluble form of Chansu developed for injection, which has been approved in China for the treatment of various forms of cancer [27, 28]. Both experimental and clinical studies have shown that cinobufacini possesses significant, broad-spectrum anticancer activity with mild side-effects [31–33, 40, 41]. However, due to limited supply of the natural raw drug material and difficulties in controlling drug composition and quality, it is impossible to develop cinobufacini as a therapeutic drug for cancer treatment. The bufadienolide glycosides cinobufagin and bufalin have been shown to be the major responsible pharmacological compounds in cinobufacini [29, 30, 35, 42]. As pure compounds, cinobufagin and bufalin have both demonstrated potent anticancer activity, which establishes them as promising candidates for developing effective anticancer drugs [35, 42]. However, they have also displayed other bioactivities including Na^+^/K^+^-ATPase inhibition [34], cardiotoxicity [43], generation of ROS [44], and inhibition of the PI3K/Akt/mTOR [45, 46], MAPK [47], Notch [48], Wnt/β-catenin [49] and STAT3 [50, 51] signaling pathways. Thus, it is difficult to use therapeutically effective doses of cinobufagin or bufalin for cancer treatment.

In this study, we showed that a sublethal dose of cinobufagin suppressed the viability of many cancer but not noncancerous cell lines. This tumor-selective cytotoxicity was blocked by the ROS inhibitor NAC, suggesting that it was resulted from cinobufagin-induced cancer-specific ROS toxicity. Oncogenic transformation is associated with elevation of oxidative stress due to increased basal ROS output [13, 14]. Thus, cancer cells are highly dependent on cellular antioxidant systems to defend against the toxicity of increased intrinsic oxidative pressure, which makes them more sensitive to additional oxidative insult or inhibition of antioxidant activities than normal cells [24, 52, 53]. Consistent with previous studies reporting that ROS-generating or antioxidant-suppressing agents can push oxidative pressure to toxic levels selectively in cancer cells [19, 22–25], our studies showed that a rapid increase in cellular ROS was induced by sublethal cinobufagin in cancer but not noncancerous cells, and ROS elevation caused cancer-specific cytotoxicity. These results suggested that the antioxidant defense capacity of the cancer cells was overwhelmed by sublethal cinobufagin-induced ROS overproduction, while the noncancerous cells were able to resist cinobufagin-induced oxidative pressure to prevent ROS overload.

Cinobufagin-induced ROS increase in the cancer cells was followed by accumulation of 8-oxoGua. The glycosylase OGG1 removes 8-oxoGua and cut the DNA strand to generate an SSB during BER [54]. SSBs are also produced directly through ROS-mediated oxidization [55]. In proliferating cells, fast moving DNA replication forks may collide with SSBs and DNA repair complexes to cause stalling and collapse of replication forks, leading to generation of replication stress and DSBs [56]. Indeed, we found that sublethal cinobufagin-induced ROS in cancer cells rapidly resulted in a marked increase in the number of DNA strand breaks including DSBs and produced intense replication stress.

DSB may activate the ATM-Chk2 DDR signaling pathway, while replication stress may result in generation of RPA-coated single-stranded DNA (ssDNA) which will activate the ATR-Chk1 DDR pathway [57, 58]. Consistently, we found that both the ATM-Chk2 and ATR-Chk1 DDR signaling pathways were strongly activated in the cinobufagin-treated cancer cells, immediately following the generation of cinobufagin-induced DSBs and replication stress. DDR signaling promotes DNA repair and induces cell cycle arrest to allow for replication fork stabilization and restart; however, if the damage or stress is not resolved in time or is unresolvable, persistent or strong DDR signaling may activate apoptosis to eliminate the cell. Prolonged cell cycle arrest and apoptosis will both result in suppression of cell viability. Here we demonstrated that in the cinobufagin-treated cancer cells, the G_2_/M checkpoint was quickly activated to induce G_2_ arrest through DDR-mediated phosphorylation of CDC25C and upregulation of p53 and p21^Cip1/Waf1^, which was followed by induction of apoptosis likely through p53-upregulated expression of pro-apoptotic proteins. Interestingly, a recent study found that arenobufagin, a minor bufadienolide isolated from Chansu, also induced apoptosis by upregulating p53 and its downstream target Noxa [59]. Together, these data indicated that sublethal cinobufagin suppressed cancer cell viability via DDR-mediated G_2_ arrest and apoptosis.

Unlike conventional DNA-damaging chemotherapies that target cancer and normal cells indiscriminately, here we showed that sublethal doses of cinobufagin were able to induce oxidative DNA damage and cytotoxicity selectively in tumor cells by exploiting a cancer vulnerability common to most oncogenically transformed cells. Within the range of the sublethal doses tested here, bufadienolides showed little or no other biological activities at the cellular level [43, 47, 51], suggesting that clinically safe and effective doses of cinobufagin can be found in the process of downstream drug development. However, cancer cells may survive genotoxic attacks by upregulating DNA repair and genome maintenance functions [57, 60]. Thus, recent years have witnessed fast development of novel anticancer agents that target DNA repair or key components of DDR, such as inhibitors of PARP1/2, ATR, ATM, Chk1, Chk2 and Wee1 [61–63]. Combining nontoxic doses of cinobufagin with these novel inhibitors is expected to produce synergistic lethality selectively in cancer cells, thereby greatly enhancing the anticancer efficacy while reducing side-effects of both drugs.

## Supplementary Information

**Additional file 1**: **Figure S1**. (A) Colony formation assay. The indicated cells were treated with PBS (control) or 100 nM CBG for 5 days. (B) Representative images of cells stained by DCFH-DA. Cells were treated by 100 nM CBG for 3 h (scale bar: 25 m). (C) Measurement of ROS levels by flow cytometry. Cells were treated by 100 nM CBG for 3 h. n.s.: not significant, *: p < 0.05, **: p < 0.01, ***: p < 0.001 vs vehicle control (n = 3).

**Additional file 2**: **Figure S2**. Cinobufagin-induced ROS overload results in oxidative DNA damage. (A) Representative images of 8-oxoG immunostaining (scale bar: 10 m) and quantification of 8-oxoG intensity in single cells. Cells were treated by 100 nM CBG for 3 h. Nuclear 8-oxoG intensity was quantified by ImageJ, at least 50 cells per sample were analyzed. (B) Representative images of alkaline comet assay (scale bar: 25 m) and quantification of tail moment in single cells. Cells were treated by 100 nM CBG for 3 h. At least 50 cells per sample were analyzed. (C) Representative images of 53BP1 immunostaining (scale bar: 25 m) and quantification of 53BP1-positive cells. Cells were treated by 100 nM CBG for 3 h. At least 5000 cells per treatment group were analyzed. n.s.: not significant, *: p < 0.05, **: p < 0.01, ***: p < 0.001, ****: p < 0.0001 vs vehicle control or NAC-treated group (n = 3).

**Additional file 3**: **Figure S3**. Cinobufagin-induced oxidative DNA damage leads to replication stress and activation of DNA damage response. (A) Representative images of gH2AX immunostaining (scale bar: 10 m) and quantification of gH2AX-positive cells. Cells were treated by 100 nM CBG for 3 h. At least 5000 cells per treatment group were analyzed. (B-D) Western blot. Cells were treated by 100 nM CBG for 3 h or the indicated times. n.s.: not significant, *: p < 0.05, **: p < 0.01, ***: p < 0.001, ****: p < 0.0001 vs vehicle control or NAC-treated group (n = 3).

## Data Availability

All data generated and analyzed during this study are included in the manuscript.

## Abbreviations

BER: Base excision repair
CBG: Cinobufagin
DAPI: 4',6-Diamidino-2-phenylindole
DCFH-DA: 2’,7’-Dichlorodihydrofluorescein diacetate
DDR: DNA damage response
DSB: Double-strand DNA break
MMP: Mitochondrial membrane potential
MTT: 3-(4,5-Dimethylthiazol-2-yl)-2,5-diphenyltetrazolium bromide
NAC: N-acetyl cysteine
PBS: Phosphate buffer saline
ROS: Reactive oxygen species
SSB: Single-strand DNA break
TUNEL: Terminal deoxynucleotidyl transferase dUTP nick end labeling

## References

[1] Pagliarini R, Shao W, Sellers WR (2015). Oncogene addiction: Pathways of therapeutic response, resistance, and road maps toward a cure. EMBO Rep.

[2] Lee YT, Tan YJ, Oon CE (2018). Molecular targeted therapy: treating cancer with specificity. Eur J Pharmacol.

[3] Huang M, Shen A, Ding J, Geng M (2014). Molecularly targeted cancer therapy: some lessons from the past decade. Trends Pharmacol Sci.

[4] Nagel R, Semenova EA, Berns A (2016). Drugging the addict: Non-oncogene addiction as a target for cancer therapy. EMBO Rep.

[5] Marusyk A, Janiszewska M, Polyak K (2020). Intratumor heterogeneity: the rosetta stone of therapy resistance. Cancer Cell.

[6] Schulze A, Harris AL (2012). How cancer metabolism is tuned for proliferation and vulnerable to disruption. Nature.

[7] Boroughs LK, DeBerardinis RJ (2015). Metabolic pathways promoting cancer cell survival and growth. Nat Cell Biol.

[8] Liberti MV, Locasale JW (2016). The warburg effect: how does it benefit cancer cells?. Trends Biochem Sci.

[9] Zecchini V, Frezza C (2017). Metabolic synthetic lethality in cancer therapy. Biochim Biophys Acta.

[10] Luengo A, Gui DY, Vander Heiden MG (2017). Targeting metabolism for cancer therapy. Cell Chem Biol.

[11] Wolpaw AJ, Dang CV (2018). Exploiting metabolic vulnerabilities of cancer with precision and accuracy. Trends Cell Biol.

[12] Schumacker PT (2006). Reactive oxygen species in cancer cells: live by the sword, die by the sword. Cancer Cell.

[13] Moloney JN, Cotter TG (2018). Ros signalling in the biology of cancer. Semin Cell Dev Biol.

[14] Saikolappan S, Kumar B, Shishodia G, Koul S, Koul HK (2019). Reactive oxygen species and cancer: a complex interaction. Cancer Lett.

[15] Irani K, Xia Y, Zweier JL, Sollott SJ, Der CJ, Fearon ER, Sundaresan M, Finkel T, Goldschmidt-Clermont PJ (1997). Mitogenic signaling mediated by oxidants in ras-transformed fibroblasts. Science.

[16] Reczek CR, Chandel NS (2015). Ros-dependent signal transduction. Curr Opin Cell Biol.

[17] Chio IIC, Tuveson DA (2017). Ros in cancer: the burning question. Trends Mol Med.

[18] Hayes JD, Dinkova-Kostova AT, Tew KD (2020). Oxidative stress in cancer. Cancer Cell.

[19] Trachootham D, Zhou Y, Zhang H, Demizu Y, Chen Z, Pelicano H, Chiao PJ, Achanta G, Arlinghaus RB, Liu J (2006). Selective killing of oncogenically transformed cells through a ros-mediated mechanism by beta-phenylethyl isothiocyanate. Cancer Cell.

[20] Nogueira V, Hay N (2013). Molecular pathways: Reactive oxygen species homeostasis in cancer cells and implications for cancer therapy. Clin Cancer Res.

[21] Liu J, Wang Z (2015). Increased oxidative stress as a selective anticancer therapy. OxidatIVE Med Cell Longev.

[22] Harris IS, Treloar AE, Inoue S, Sasaki M, Gorrini C, Lee KC, Yung KY, Brenner D, Knobbe-Thomsen CB, Cox MA (2015). Glutathione and thioredoxin antioxidant pathways synergize to drive cancer initiation and progression. Cancer Cell.

[23] Ding Y, Wang H, Niu J, Luo M, Gou Y, Miao L, Zou Z, Cheng Y (2016). Induction of ros overload by alantolactone prompts oxidative DNA damage and apoptosis in colorectal cancer cells. Int J Mol Sci.

[24] AbdulSalam SF, Thowfeik FS, Merino EJ (2016). Excessive reactive oxygen species and exotic DNA lesions as an exploitable liability. Biochemistry.

[25] Wang H, Zhang S, Song L, Qu M, Zou Z (2020). Synergistic lethality between parp-trapping and alantolactone-induced oxidative DNA damage in homologous recombination-proficient cancer cells. Oncogene.

[26] Qi J, Tan CK, Hashimi SM, Zulfiker AH, Good D, Wei MQ (2014). Toad glandular secretions and skin extractions as anti-inflammatory and anticancer agents. Evid based Complement Altern Med eCAM.

[27] Qi J, Zulfiker A, Li C, Good D, Wei M (2018). The development of toad toxins as potential therapeutic agents. Toxins.

[28] Qi F, Li A, Inagaki Y, Kokudo N, Tamura S, Nakata M, Tang W (2011). Antitumor activity of extracts and compounds from the skin of the toad bufo bufo gargarizans cantor. Int Immunopharmacol.

[29] Li C, Hashimi SM, Cao S, Mellick AS, Duan W, Good D, Wei MQ (2013). The mechanisms of chansu in inducing efficient apoptosis in colon cancer cells. Evid Based Complement Altern Med eCAM.

[30] Lee S, Lee Y, Choi YJ, Han KS, Chung HW (2014). Cyto-/genotoxic effects of the ethanol extract of chan su, a traditional chinese medicine, in human cancer cell lines. J Ethnopharmacol.

[31] Meng Z, Yang P, Shen Y, Bei W, Zhang Y, Ge Y, Newman RA, Cohen L, Liu L, Thornton B (2009). Pilot study of huachansu in patients with hepatocellular carcinoma, nonsmall-cell lung cancer, or pancreatic cancer. Cancer.

[32] Zhou B, Wu F, Yuan L, Miao Z, Zhu S (2015). Is huachansu beneficial in treating advanced non-small-cell lung cancer? Evidence from a meta-analysis of its efficacy combined with chemotherapy. Evid Based Complement Altern Med eCAM.

[33] Yang T, Shi R, Chang L, Tang K, Chen K, Yu G, Tian Y, Guo Y, He W, Song X (2015). Huachansu suppresses human bladder cancer cell growth through the fas/fasl and tnf- alpha/tnfr1 pathway in vitro and in vivo. J Exp Clin Cancer Res CR.

[34] Sousa LQ, Machado KD, Oliveira SF, Araujo LD, Moncao-Filho ED, Melo-Cavalcante AA, Vieira-Junior GM, Ferreira PM (2017). Bufadienolides from amphibians: A promising source of anticancer prototypes for radical innovation, apoptosis triggering and na+/k+-atpase inhibition. Toxicon.

[35] Wei X, Si N, Zhang Y, Zhao H, Yang J, Wang H, Wang L, Han L, Bian B (2017). Evaluation of bufadienolides as the main antitumor components in cinobufacin injection for liver and gastric cancer therapy. PLoS ONE.

[36] Yang Q, Zhou X, Zhang M, Bi L, Miao S, Cao W, Xie Y, Sun J, Tang H, Li Y (2015). Angel of human health: current research updates in toad medicine. Am J Transl Res.

[37] Baek SH, Kim C, Lee JH, Nam D, Lee J, Lee SG, Chung WS, Jang HJ, Kim SH, Ahn KS (2015). Cinobufagin exerts anti-proliferative and pro-apoptotic effects through the modulation ros-mediated mapks signaling pathway. Immunopharmacol Immunotoxicol.

[38] Ma K, Zhang C, Huang MY, Li WY, Hu GQ (2016). Cinobufagin induces autophagy-mediated cell death in human osteosarcoma u2os cells through the ros/jnk/p38 signaling pathway. Oncol Rep.

[39] Struthers L, Patel R, Clark J, Thomas S (1998). Direct detection of 8-oxodeoxyguanosine and 8-oxoguanine by avidin and its analogues. Anal Biochem.

[40] Ma L, Song B, Jin H, Pi J, Liu L, Jiang J, Cai J (2012). Cinobufacini induced mda-mb-231 cell apoptosis-associated cell cycle arrest and cytoskeleton function. Bioorg Med Chem Lett.

[41] Yin JH, Zhu XY, Shi WD, Liu LM (2014). Huachansu injection inhibits metastasis of pancreatic cancer in mice model of human tumor xenograft. BMC Complement Altern Med.

[42] Deng L-J, Li Y, Qi M, Liu J-S, Wang S, Hu L-J, Lei Y-H, Jiang R-W, Chen W-M, Qi Q (2020). Molecular mechanisms of bufadienolides and their novel strategies for cancer treatment. Eur J Pharmacol.

[43] Li M, Wang XJ, Zhao Q, Wang JX, Xing HY, Zhang YZ, Zhang XX, Zhi YY, Li H, Ma J (2020). Bufalin-induced cardiotoxicity: new findings into mechanisms. Chin J Nat Med.

[44] Pan L, Nie L, Yao S, Bi A, Ye Y, Wu Y, Tan Z, Wu Z (2020). Bufalin exerts antitumor effects in neuroblastoma via the induction of reactive oxygen speciesmediated apoptosis by targeting the electron transport chain. Int J Mol Med.

[45] Zhang G, Wang C, Sun M, Li J, Wang B, Jin C, Hua P, Song G, Zhang Y, Nguyen LL (2016). Cinobufagin inhibits tumor growth by inducing intrinsic apoptosis through akt signaling pathway in human nonsmall cell lung cancer cells. Oncotarget.

[46] Wang H, Zhang C, Xu L, Zang K, Ning Z, Jiang F, Chi H, Zhu X, Meng Z (2016). Bufalin suppresses hepatocellular carcinoma invasion and metastasis by targeting hif-1alpha via the pi3k/akt/mtor pathway. Oncotarget.

[47] Xu ZW, Wang FM, Gao MJ, Chen XY, Shan NN, Cheng SX, Mai X, Zala GH, Hu WL, Xu RC (2011). Cardiotonic steroids attenuate erk phosphorylation and generate cell cycle arrest to block human hepatoma cell growth. J Steroid Biochem Mol Biol.

[48] Cao Y, Yu L, Dai G, Zhang S, Zhang Z, Gao T, Guo W (2017). Cinobufagin induces apoptosis of osteosarcoma cells through inactivation of notch signaling. Eur J Pharmacol.

[49] Yu Z, Feng H, Sun X, Zhuo Y, Li M, Zhou Z, Huang L, Jiang Y, Zhu X, Zhang X (2018). Bufalin suppresses hepatocarcinogenesis by targeting β-catenin/tcf signaling via cell cycle-related kinase. Sci Rep.

[50] Yang L, Zhou F, Zhuang Y, Liu Y, Xu L, Zhao H, Xiang Y, Dai X, Liu Z, Huang X (2021). Acetyl-bufalin shows potent efficacy against non-small-cell lung cancer by targeting the cdk9/stat3 signalling pathway. Br J Cancer.

[51] Bai Y, Wang X, Cai M, Ma C, Xiang Y, Hu W, Zhou B, Zhao C, Dai X, Li X (2021). Cinobufagin suppresses colorectal cancer growth via stat3 pathway inhibition. Am J Cancer Res.

[52] Harris I, DeNicola G (2020). The complex interplay between antioxidants and ros in cancer. Trends Cell Biol.

[53] Gorrini C, Harris IS, Mak TW (2013). Modulation of oxidative stress as an anticancer strategy. Nat Rev Drug Discovery.

[54] Markkanen E (2017). Not breathing is not an option: how to deal with oxidative DNA damage. DNA Repair.

[55] Davalli P, Marverti G, Lauriola A, D'Arca D (2018). Targeting oxidatively induced DNA damage response in cancer: opportunities for novel cancer therapies. Oxid Med Cell Longev.

[56] Hanzlikova H, Caldecott KW (2019). Perspectives on parps in s phase. Trends Genet.

[57] Ciccia A, Elledge SJ (2010). The DNA damage response: making it safe to play with knives. Mol Cell.

[58] Blackford AN, Jackson SP (2017). Atm, atr, and DNA-pk: the trinity at the heart of the DNA damage response. Mol Cell.

[59] Ma L, Zhu Y, Fang S, Long H, Liu X, Liu Z (2017). Arenobufagin induces apoptotic cell death in human non-small-cell lung cancer cells via the noxa-related pathway. Molecules (Basel, Switzerland).

[60] Roos WP, Thomas AD, Kaina B (2016). DNA damage and the balance between survival and death in cancer biology. Nat Rev Cancer.

[61] Pilié PG, Tang C, Mills GB, Yap TA (2019). State-of-the-art strategies for targeting the DNA damage response in cancer. Nat Rev Clin Oncol.

[62] Lecona E, Fernandez-Capetillo O (2018). Targeting atr in cancer. Nat Rev Cancer.

[63] Nickoloff J, Jones D, Lee S, Williamson E, Hromas R (2017). Drugging the cancers addicted to DNA repair. J Natl Cancer Inst.

