# Supplementary material for: Cinobufagin-induced DNA damage response activates G2/M checkpoint and apoptosis to cause selective cytotoxicity in cancer cells
Source: Cancer Cell Int. 2021 Aug 23;21:446. doi: 10.1186/s12935-021-02150-0 (PMC8381584; doi:10.1186/s12935-021-02150-0)

**Figure S1.** (**A**) Colony formation assay. The indicated cells were treated with PBS (control) or 100 nM CBG for 5 days. (**B**) Representative images of cells stained by DCFH-DA. Cells were treated by 100 nM CBG for 3 h (scale bar: 25 μm). (**C**) Measurement of ROS levels by flow cytometry. Cells were treated by 100 nM CBG for 3 h. n.s.: not significant, *: *p* < 0.05, **: *p* < 0.01, ***: *p* < 0.001 vs vehicle control (n = 3).

**A**


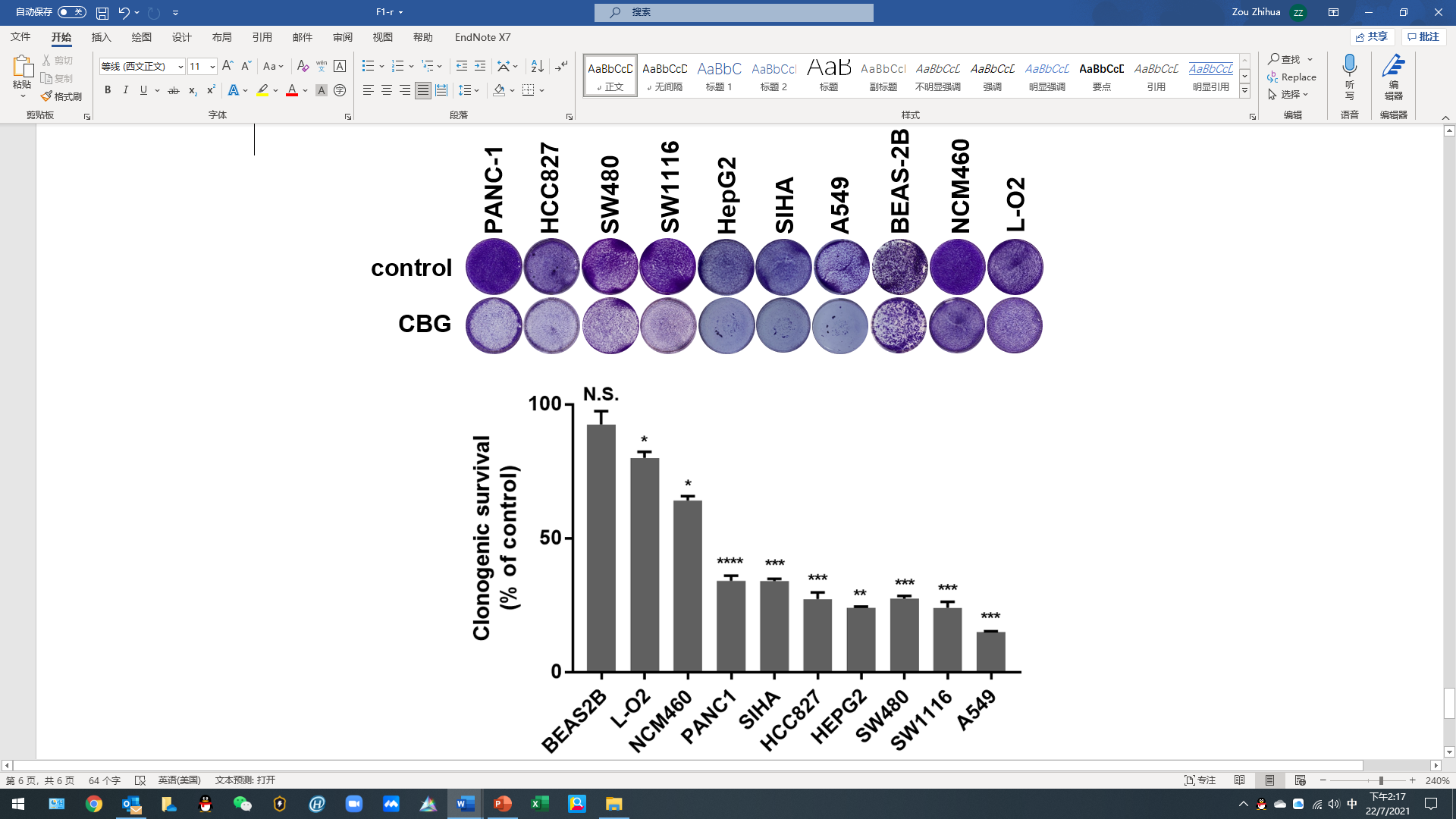

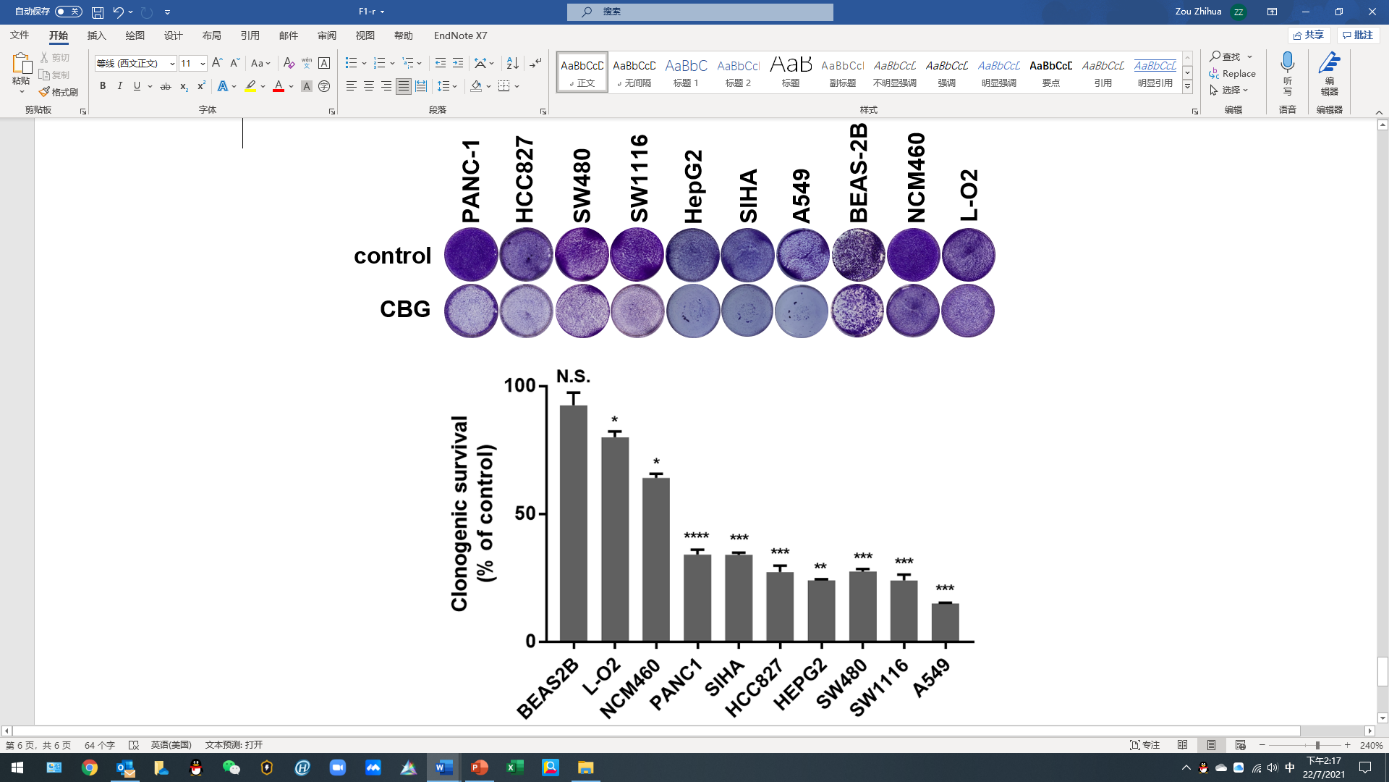


**B**


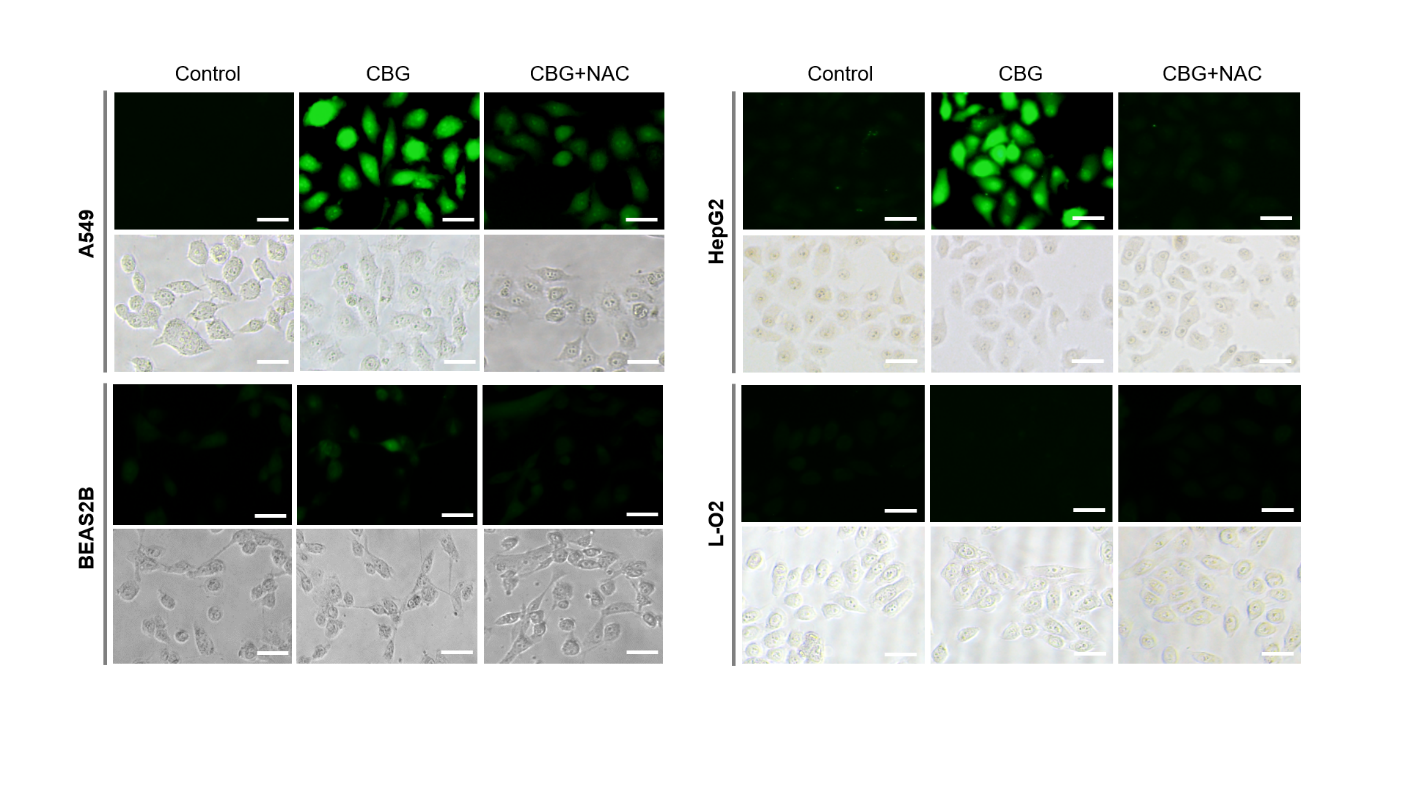

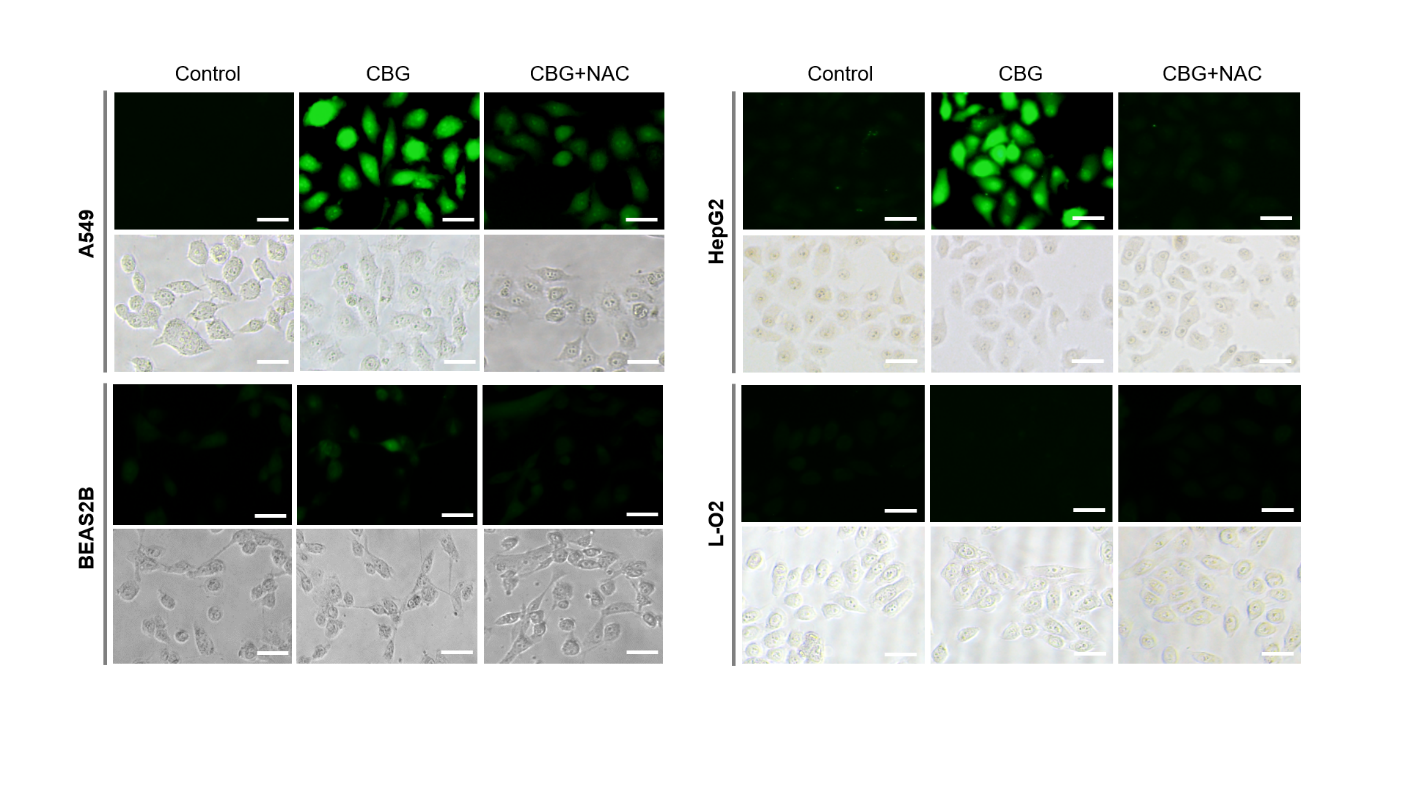


**C**

**A549**


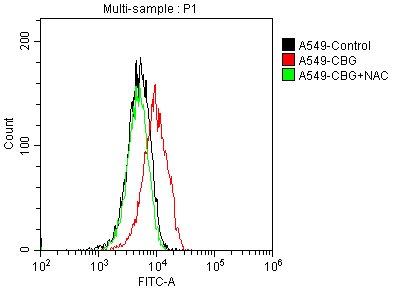

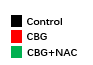

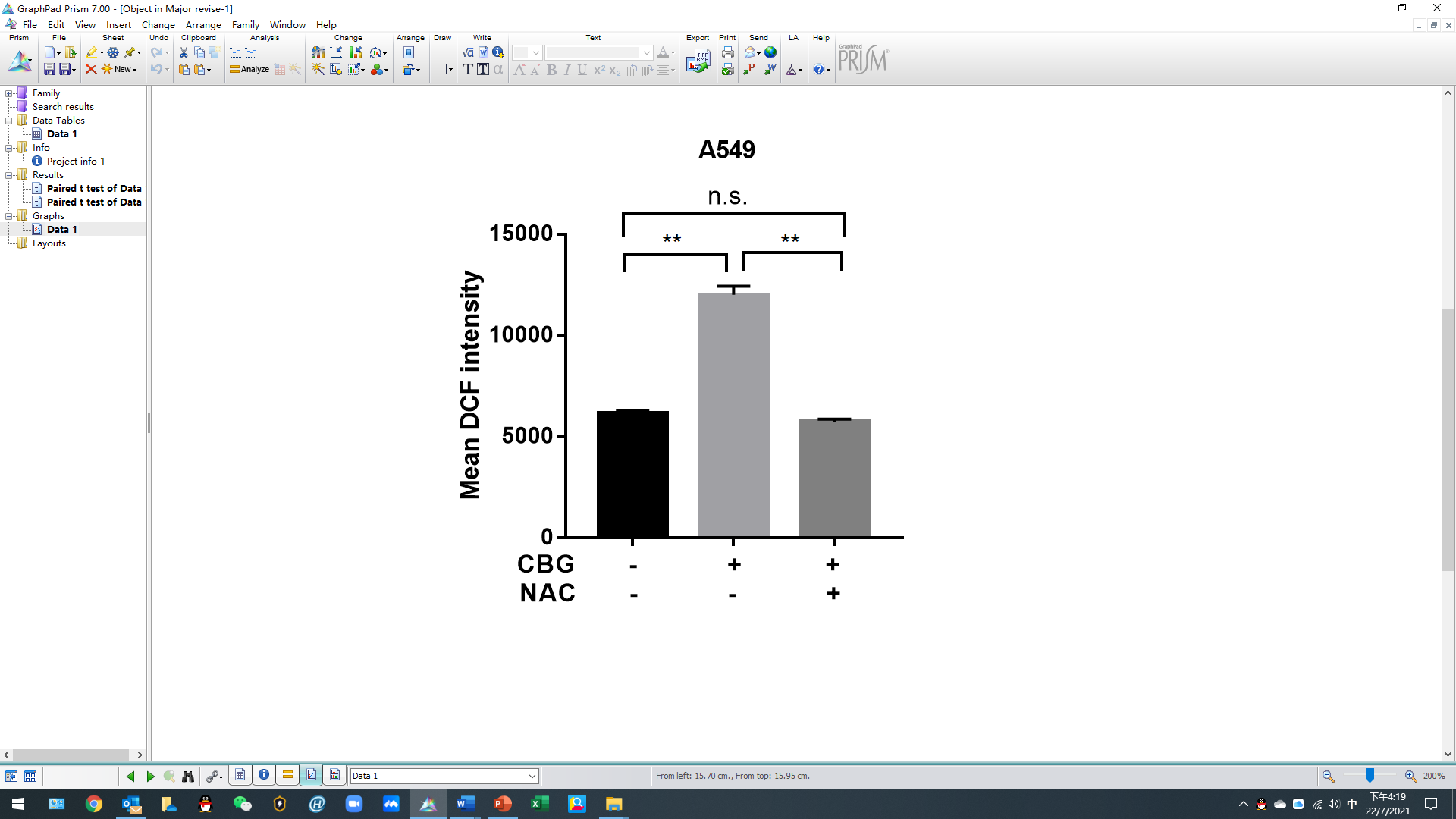


**BEAS-2B**


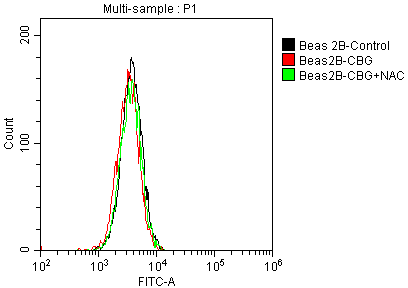

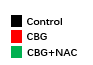

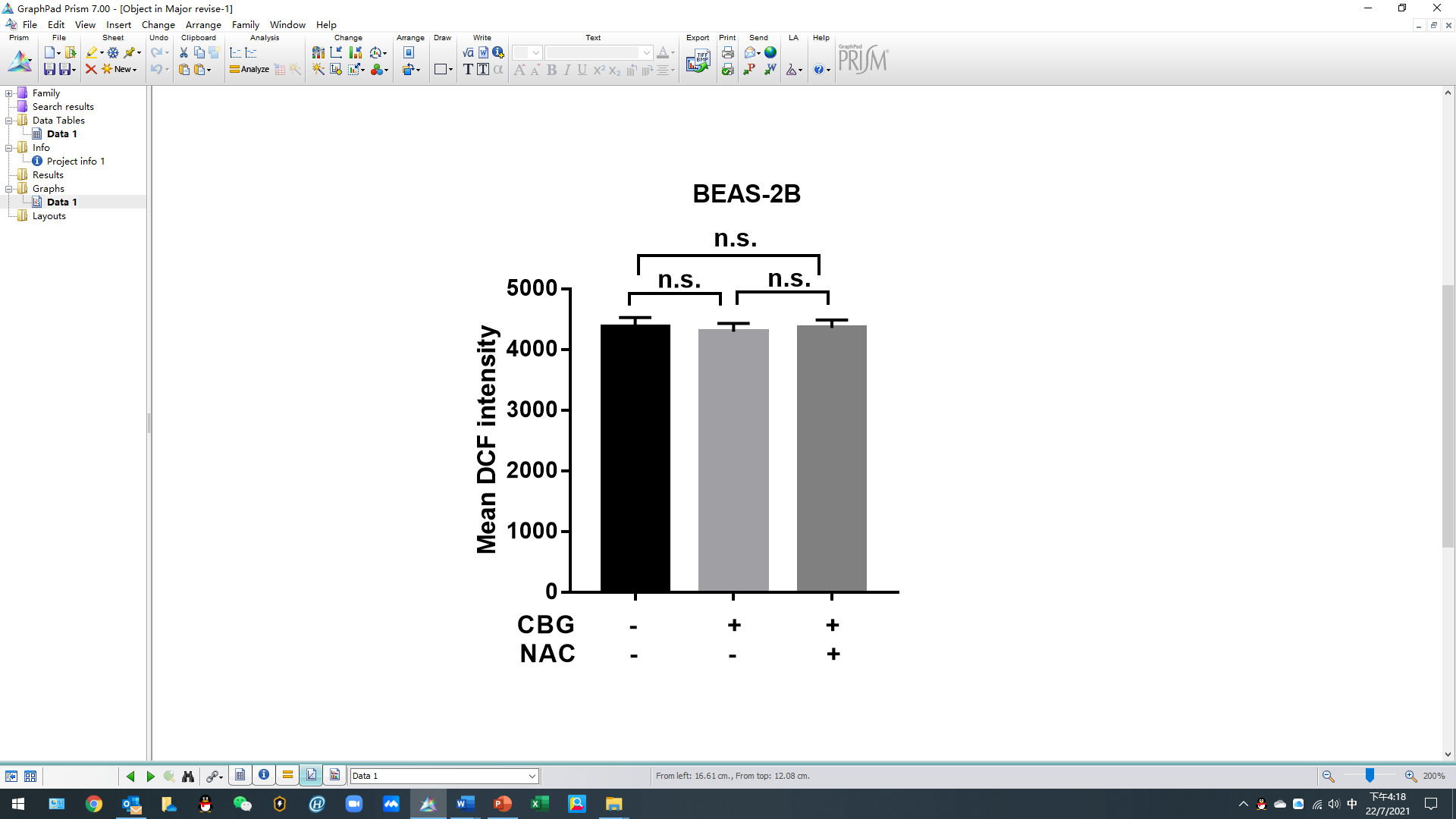


**HepG2**


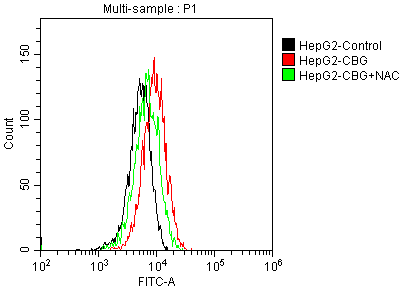

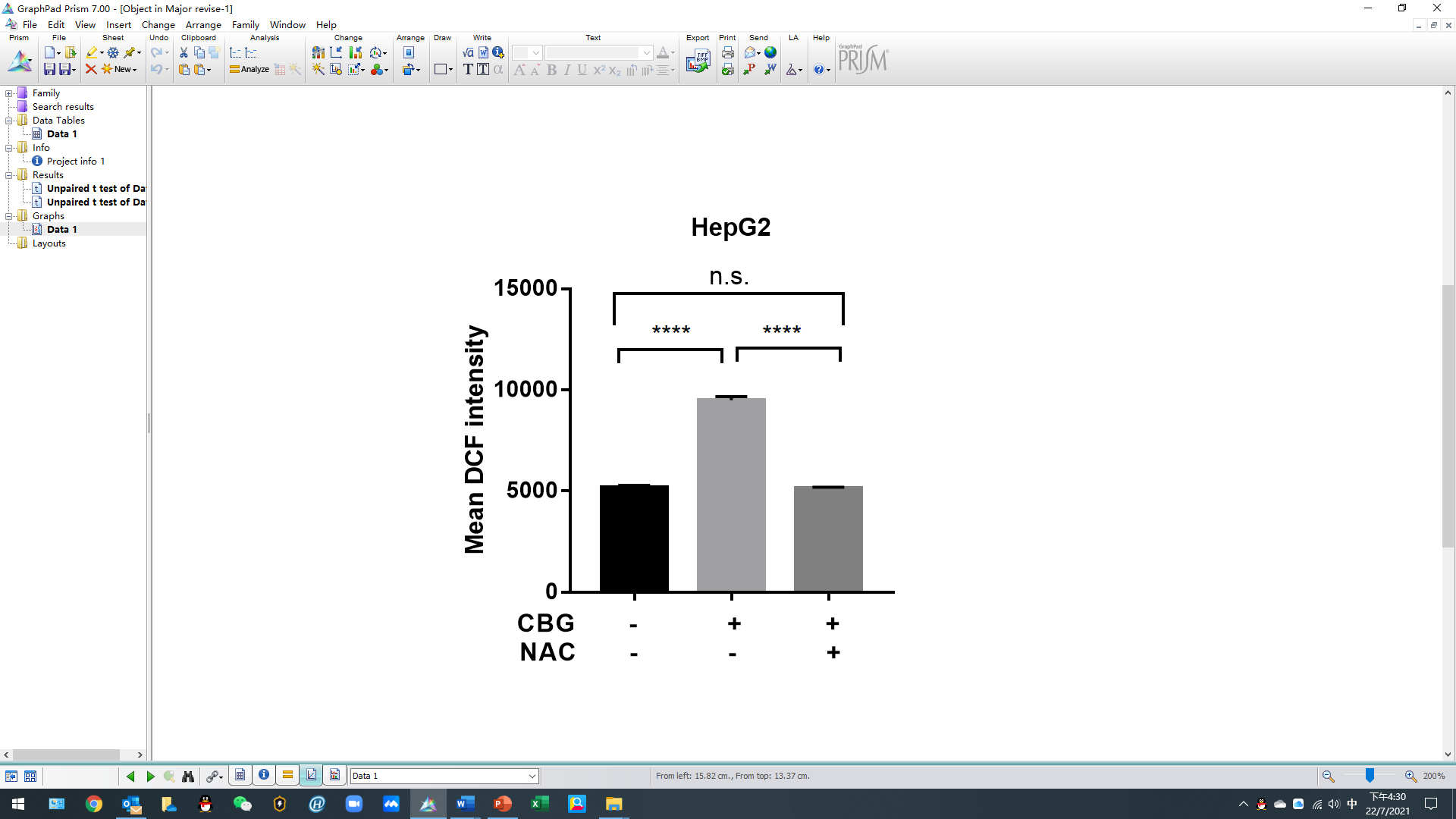


**L-O2**


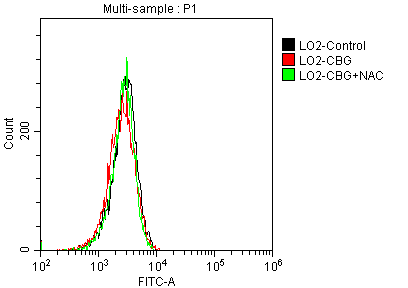

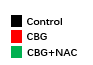

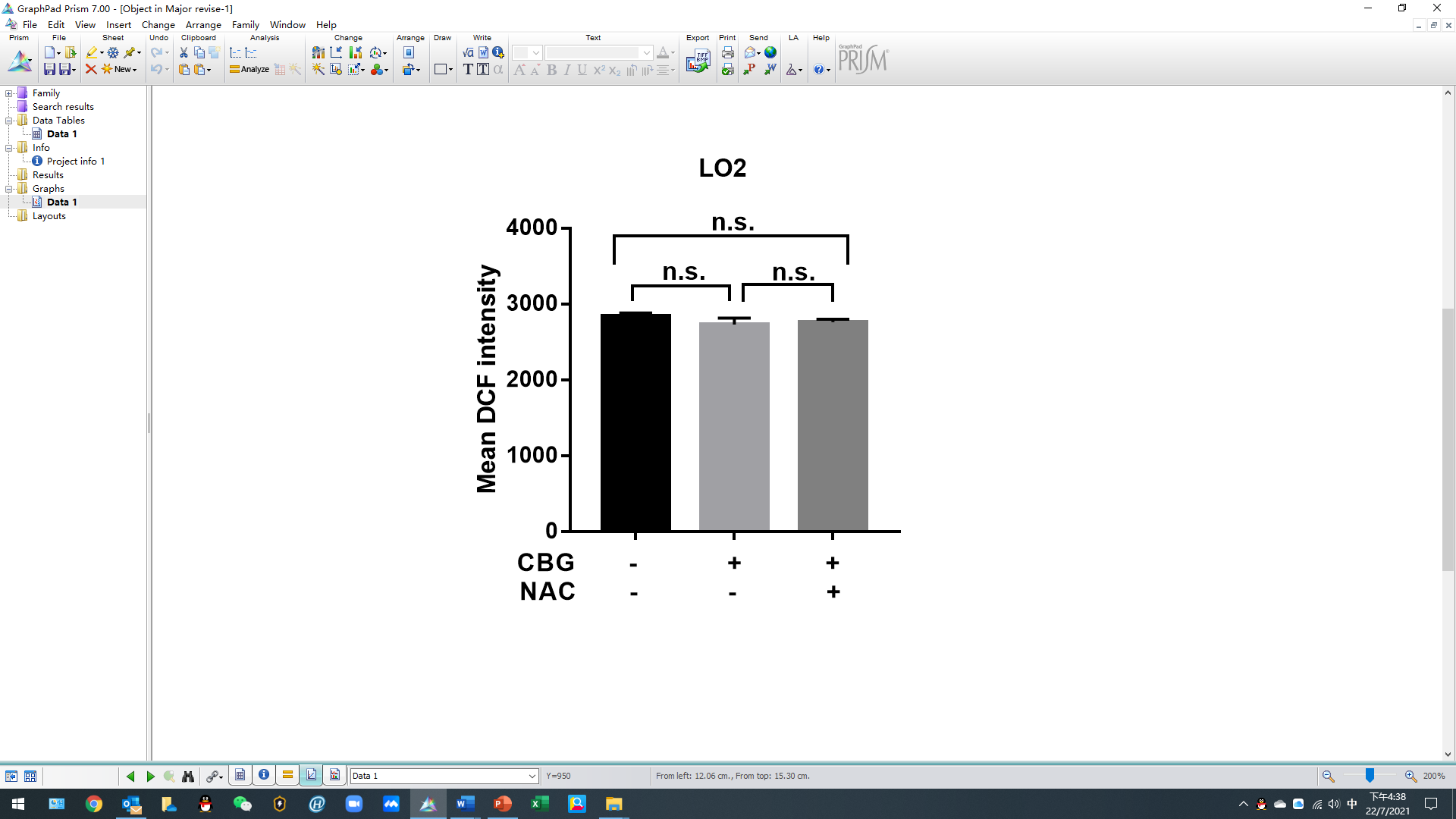

Supplement: Supplementary file 1 — Additional file 1: Figure S1. (A) Colony formation assay. The indicated cells were treated with PBS (control) or 100 nM CBG for 5 days. (B) Representative images of cells stained by DCFH-DA. Cells were treated by 100 nM CBG for 3 h (scale bar: 25 m). (C) Measurement of ROS levels by flow cytometry. Cells were treated by 100 nM CBG for 3 h. n.s.: not significant, *: p < 0.05, **: p < 0.01, ***: p < 0.001 vs vehicle control (n = 3). [file 12935_2021_2150_MOESM1_ESM.docx]
