# Supplementary material for: Cinobufagin-induced DNA damage response activates G2/M checkpoint and apoptosis to cause selective cytotoxicity in cancer cells
Source: Cancer Cell Int. 2021 Aug 23;21:446. doi: 10.1186/s12935-021-02150-0 (PMC8381584; doi:10.1186/s12935-021-02150-0)
Supplement: Supplementary file 2 — Additional file 2: Figure S2. Cinobufagin-induced ROS overload results in oxidative DNA damage. (A) Representative images of 8-oxoG immunostaining (scale bar: 10 m) and quantification of 8-oxoG intensity in single cells. Cells were treated by 100 nM CBG for 3 h. Nuclear 8-oxoG intensity was quantified by ImageJ, at least 50 cells per sample were analyzed. (B) Representative images of alkaline comet assay (scale bar: 25 m) and quantification of tail moment in single cells. Cells were treated by 100 nM CBG for 3 h. At least 50 cells per sample were analyzed. (C) Representative images of 53BP1 immunostaining (scale bar: 25 m) and quantification of 53BP1-positive cells. Cells were treated by 100 nM CBG for 3 h. At least 5000 cells per treatment group were analyzed. n.s.: not significant, *: p < 0.05, **: p < 0.01, ***: p < 0.001, ****: p < 0.0001 vs vehicle control or NAC-treated group (n = 3). [file 12935_2021_2150_MOESM2_ESM.docx]

**A**


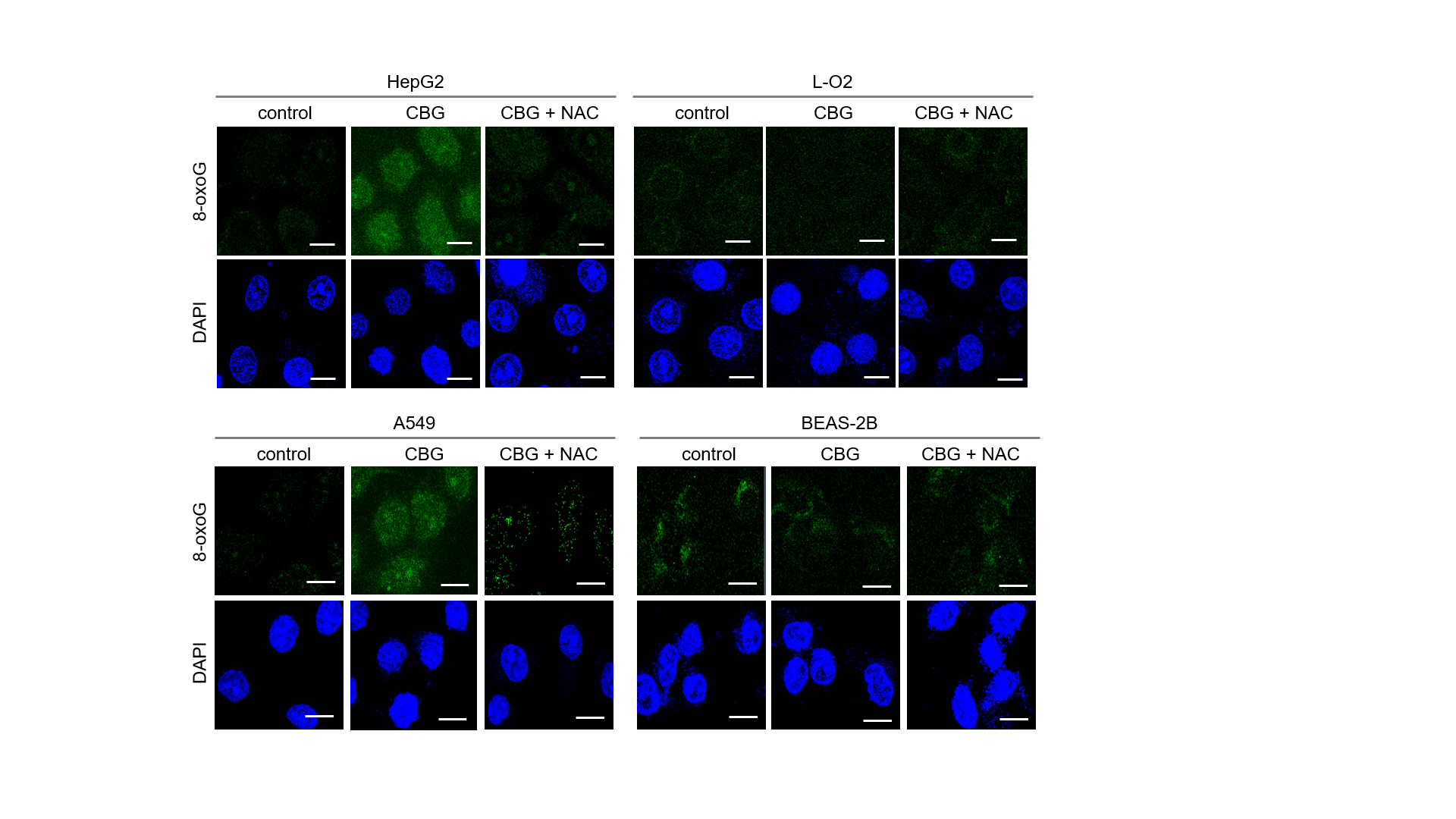

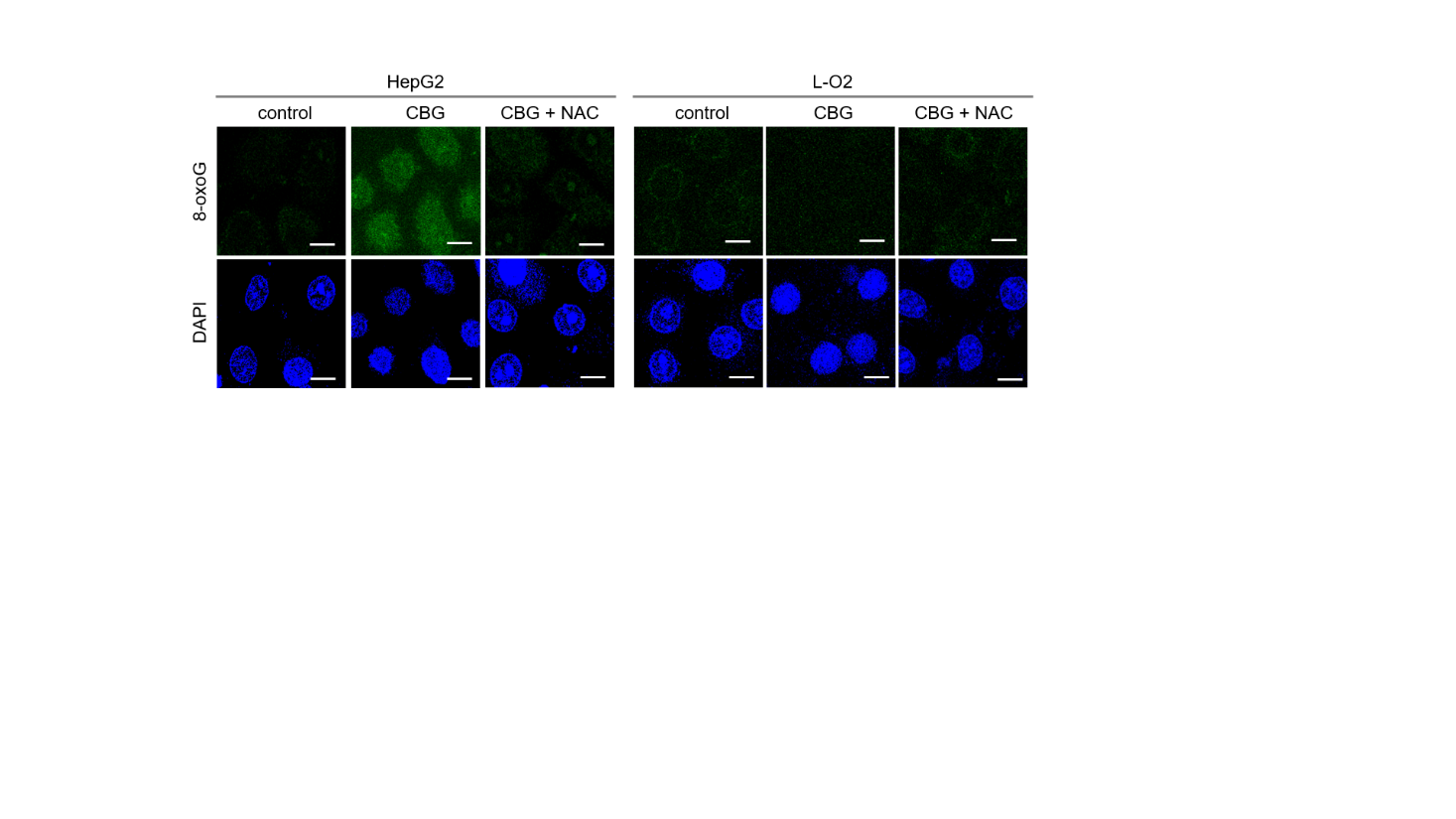

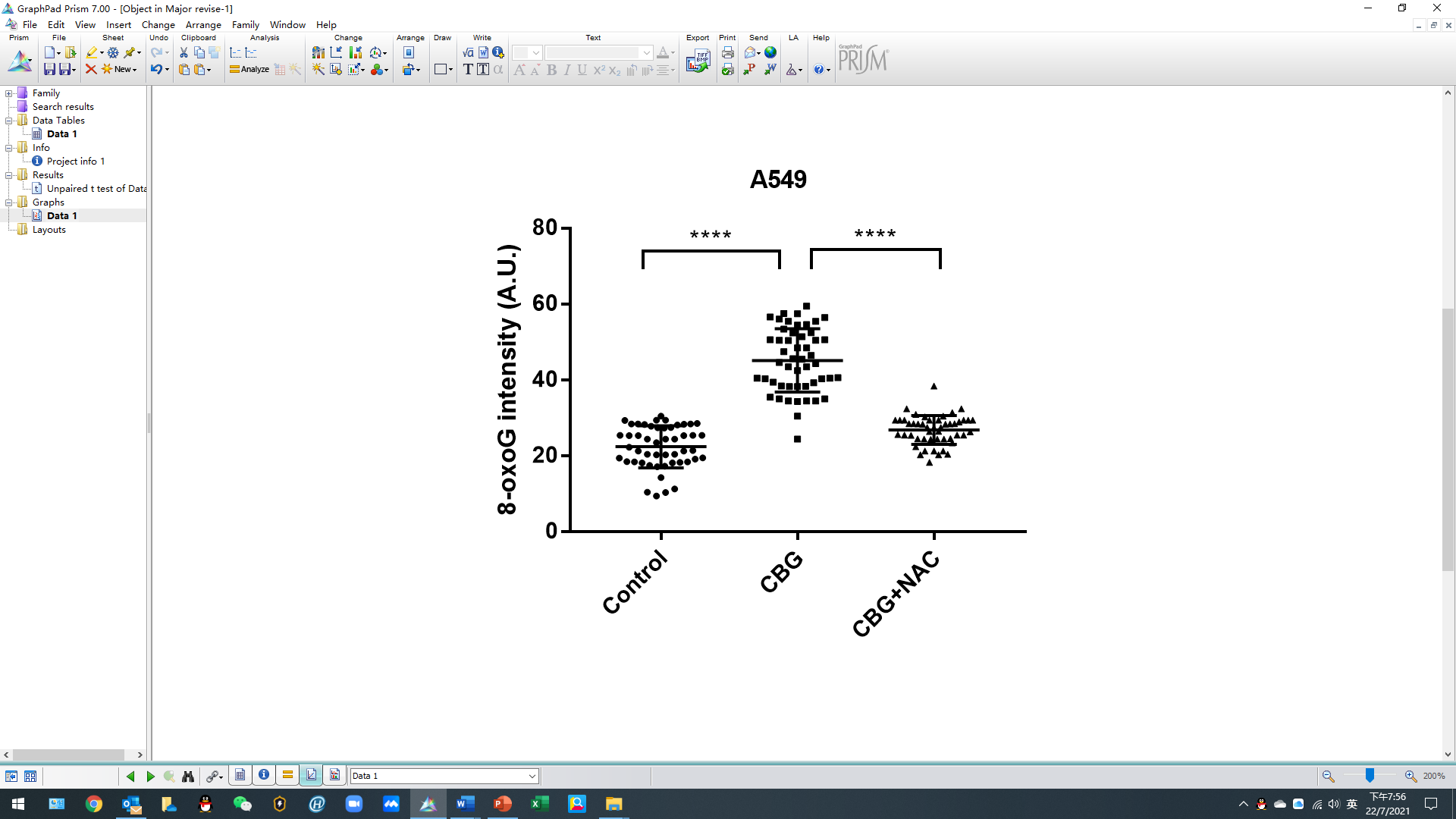


**A549**


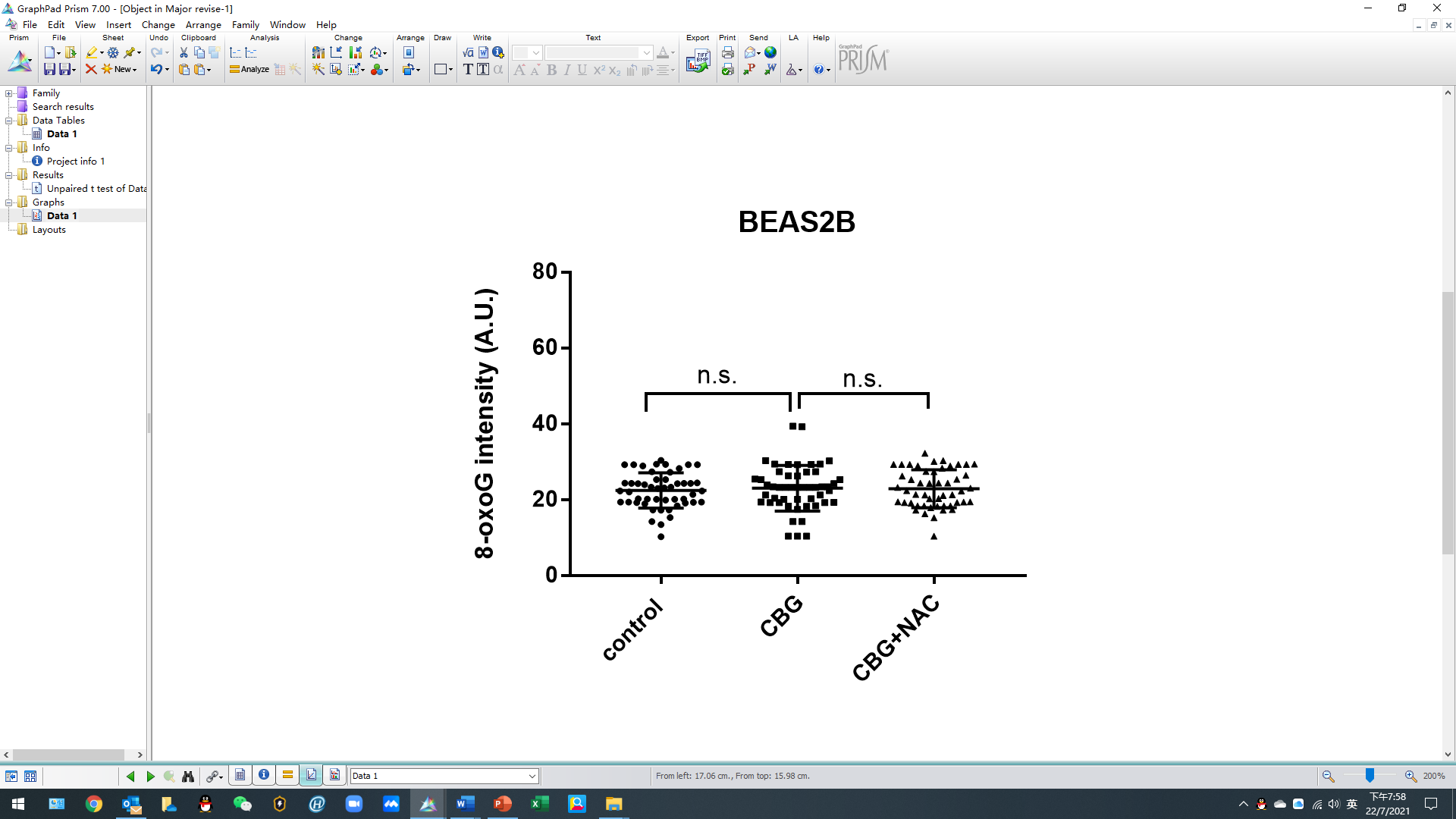


**BEAS-2B**


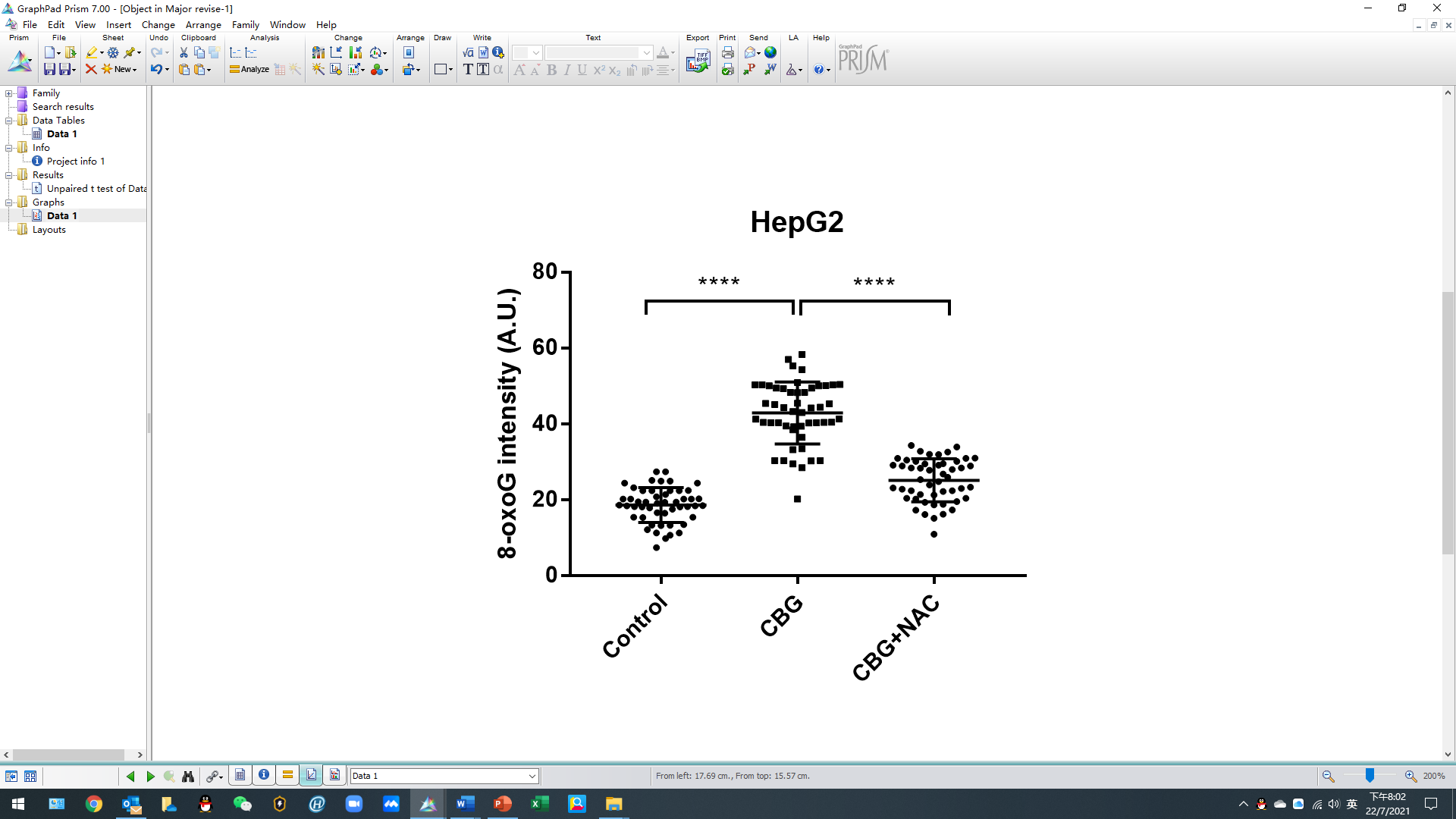


**HepG2**


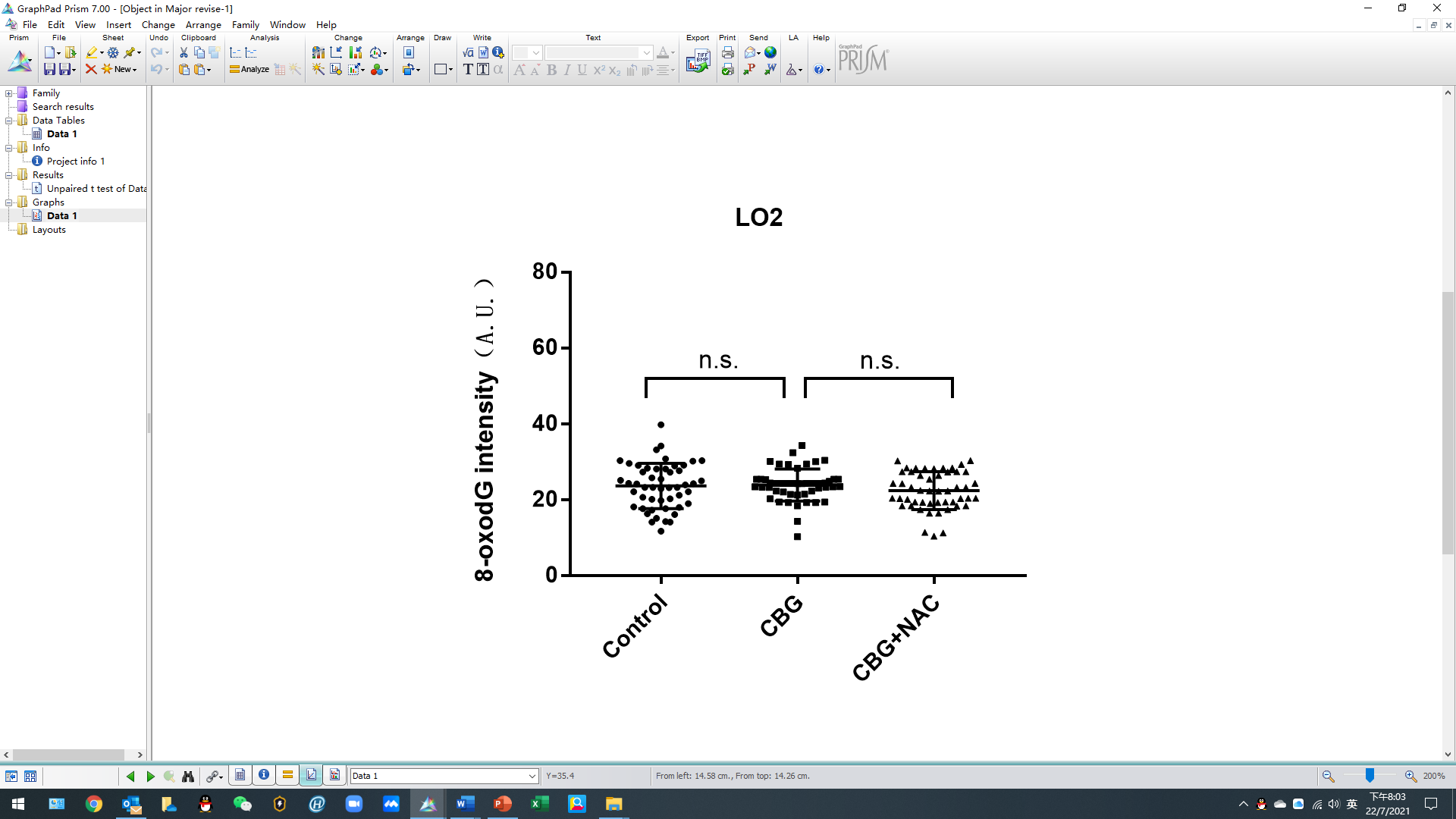


**L-O2**

**B**


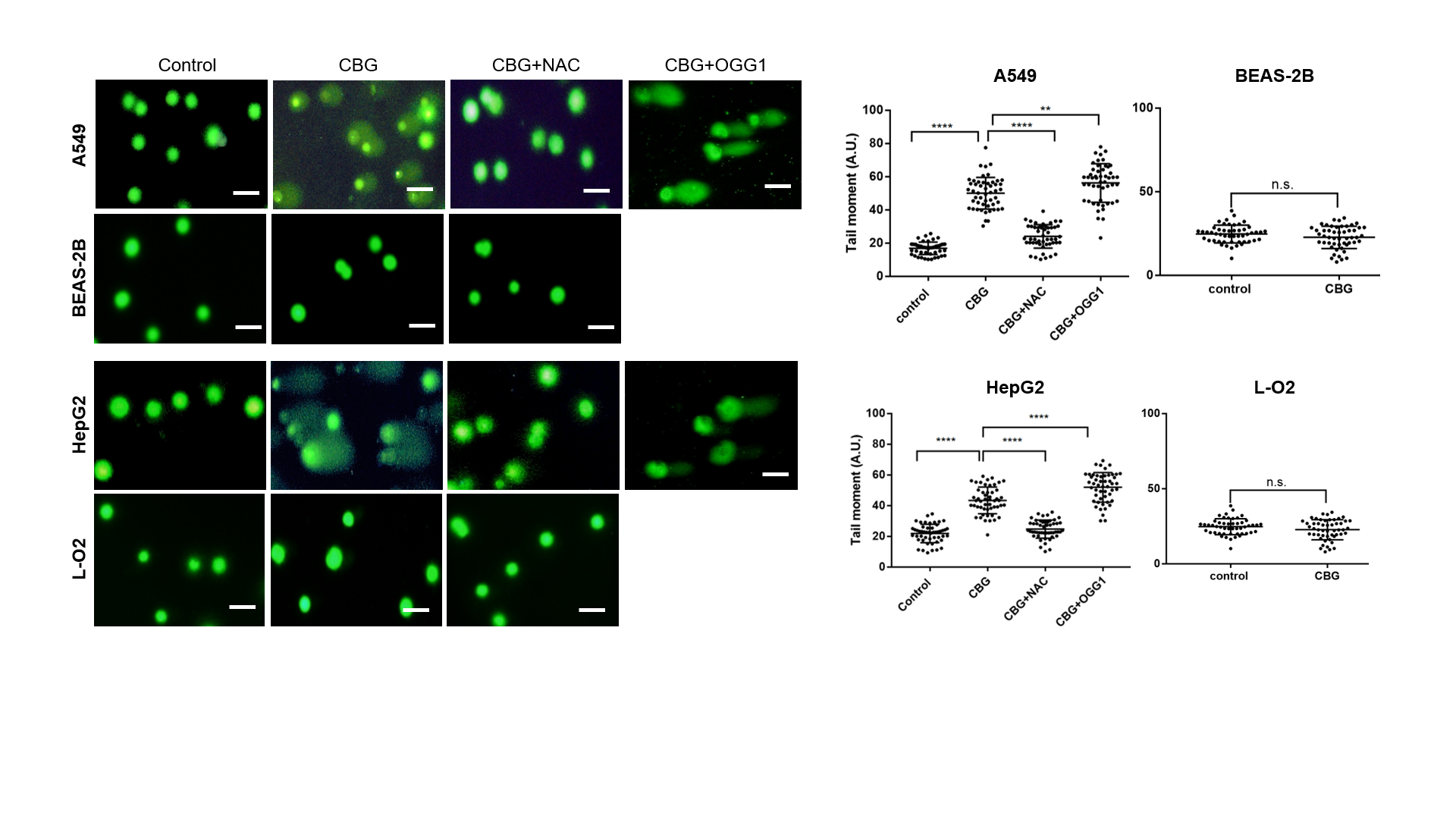

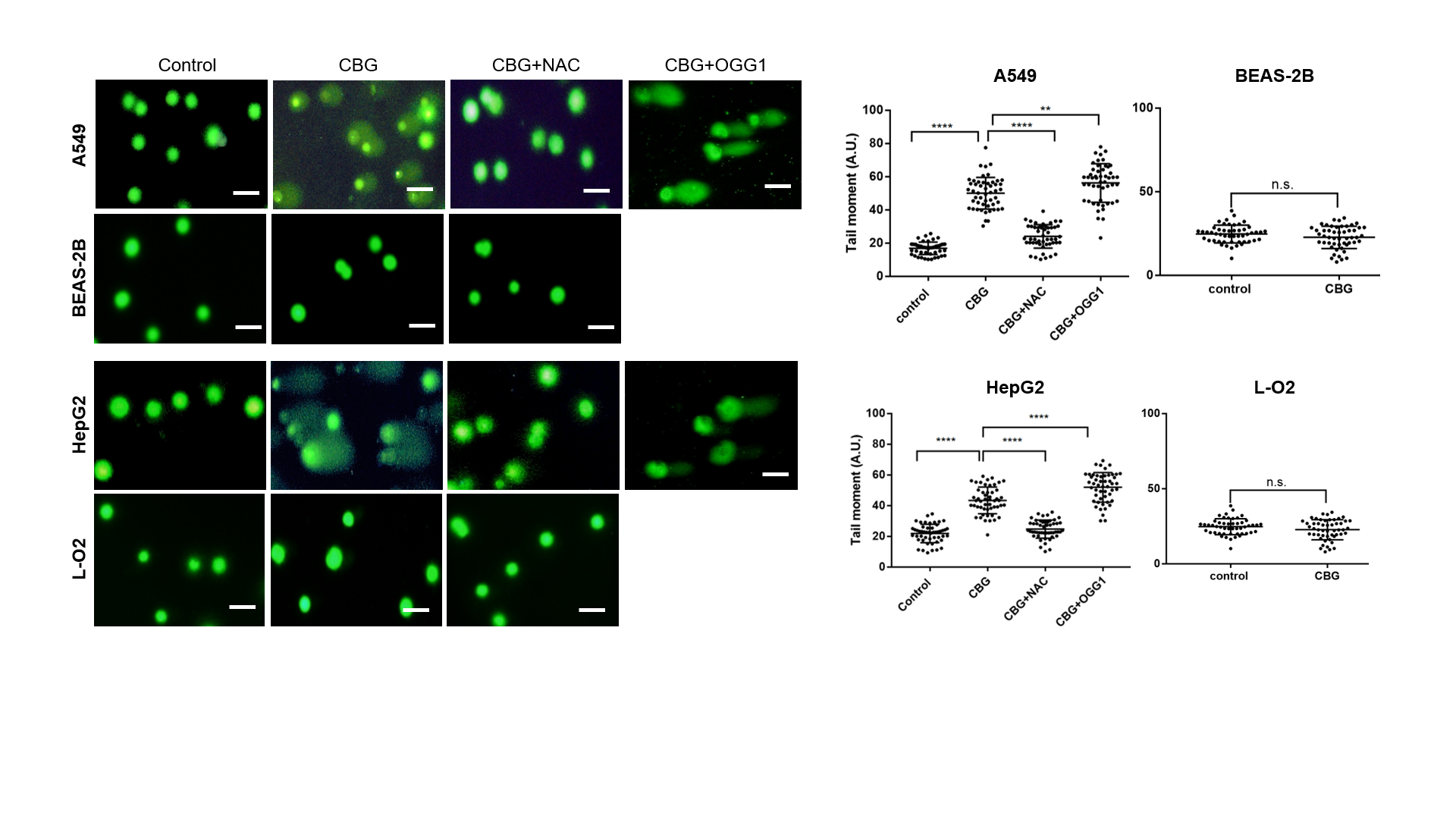


**C**


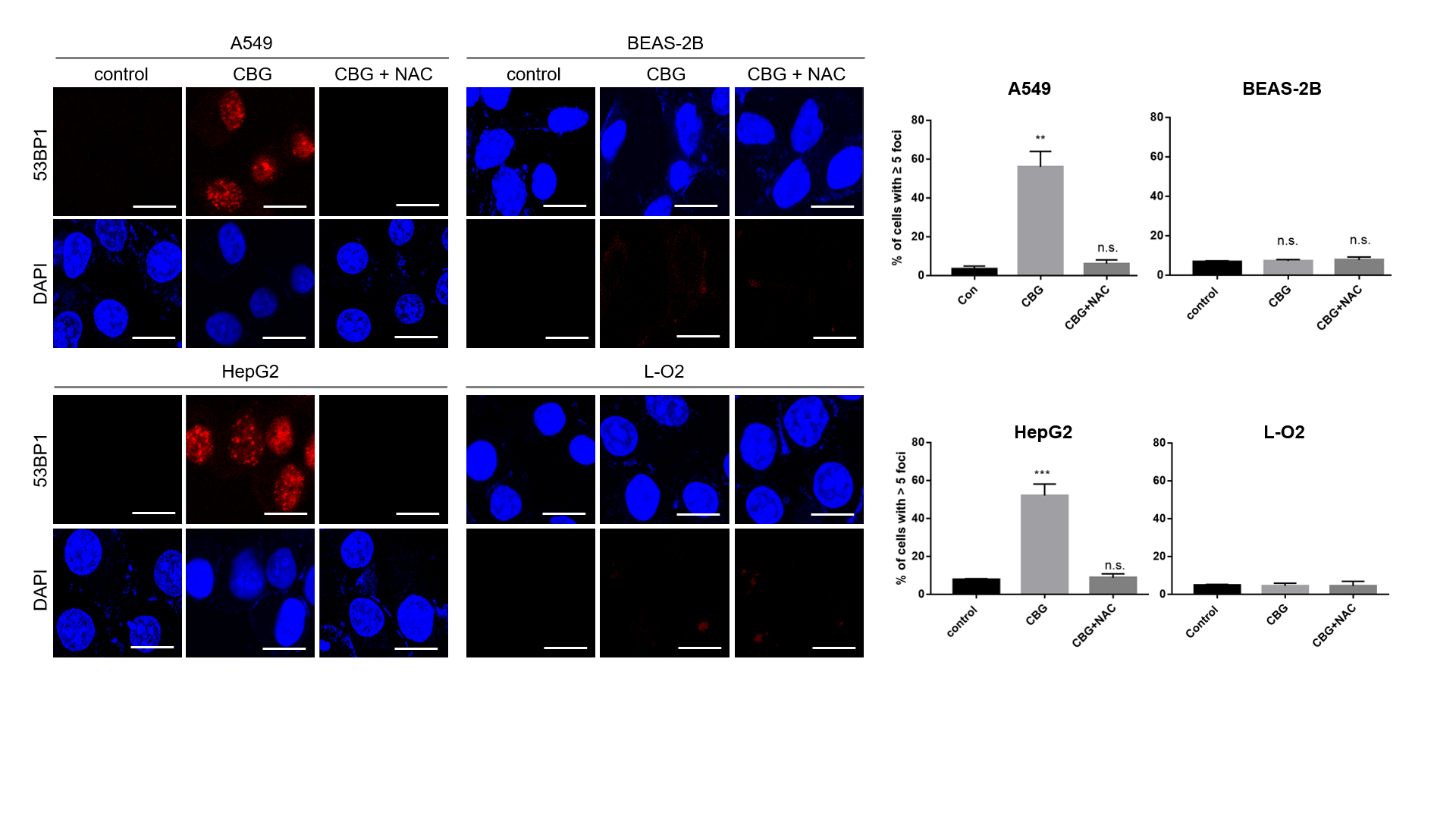

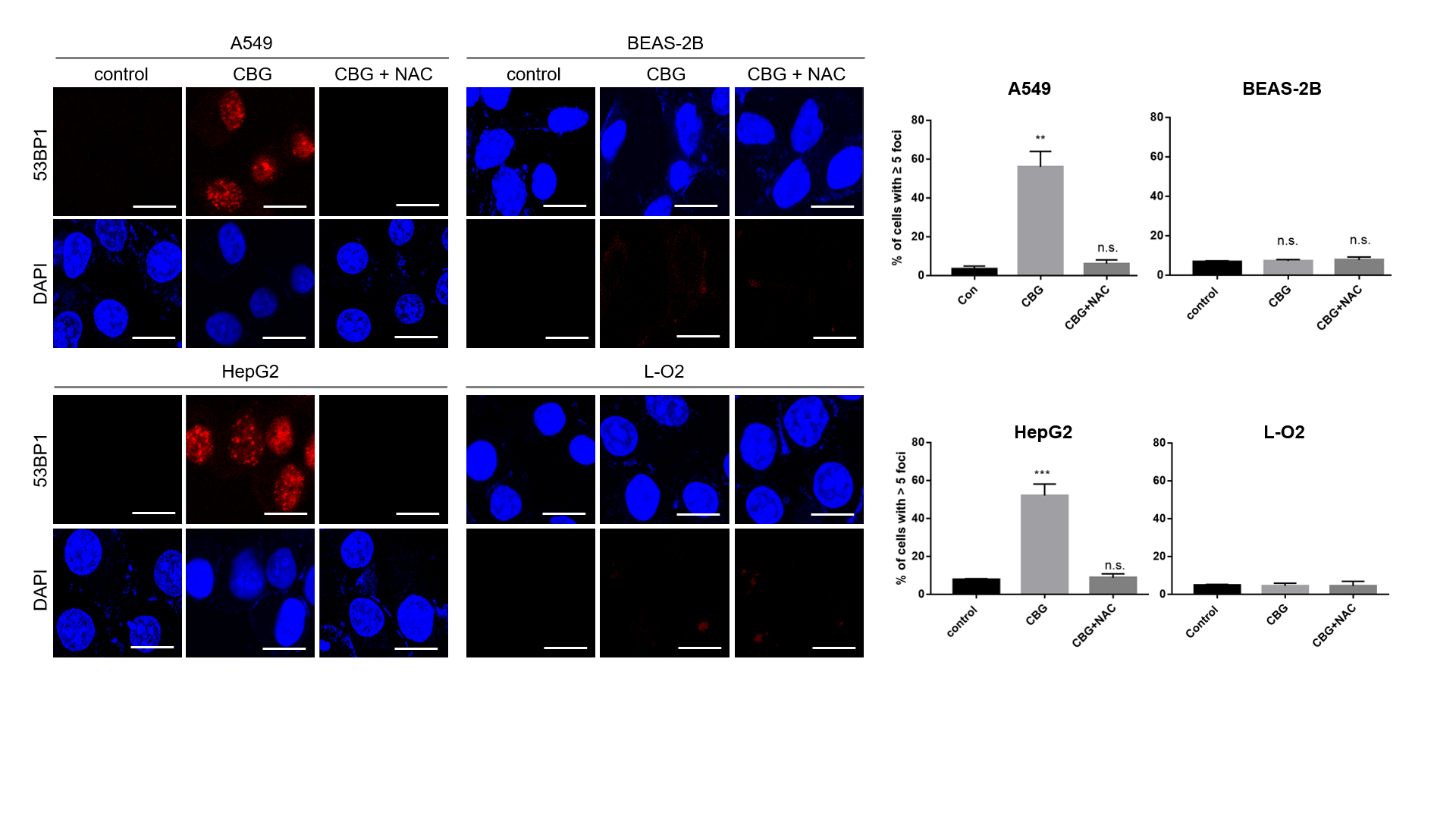


**Figure S2.** Cinobufagin-induced ROS overload results in oxidative DNA damage. (**A**) Representative images of 8-oxoG immunostaining (scale bar: 10 μm) and quantification of 8-oxoG intensity in single cells. Cells were treated by 100 nM CBG for 3 h. Nuclear 8-oxoG intensity was quantified by ImageJ, at least 50 cells per sample were analyzed. (**B**) Representative images of alkaline comet assay (scale bar: 25 μm) and quantification of tail moment in single cells. Cells were treated by 100 nM CBG for 3 h. At least 50 cells per sample were analyzed. (**C**) Representative images of 53BP1 immunostaining (scale bar: 25 μm) and quantification of 53BP1-positive cells. Cells were treated by 100 nM CBG for 3 h. At least 5000 cells per treatment group were analyzed. n.s.: not significant, *: *p* < 0.05, **: *p* < 0.01, ***: *p* < 0.001, ****: *p* < 0.0001 vs vehicle control or NAC-treated group (n = 3).
