# Supplementary material for: Cinobufagin-induced DNA damage response activates G2/M checkpoint and apoptosis to cause selective cytotoxicity in cancer cells
Source: Cancer Cell Int. 2021 Aug 23;21:446. doi: 10.1186/s12935-021-02150-0 (PMC8381584; doi:10.1186/s12935-021-02150-0)
Supplement: Supplementary file 3 — Additional file 3: Figure S3. Cinobufagin-induced oxidative DNA damage leads to replication stress and activation of DNA damage response. (A) Representative images of gH2AX immunostaining (scale bar: 10 m) and quantification of gH2AX-positive cells. Cells were treated by 100 nM CBG for 3 h. At least 5000 cells per treatment group were analyzed. (B-D) Western blot. Cells were treated by 100 nM CBG for 3 h or the indicated times. n.s.: not significant, *: p < 0.05, **: p < 0.01, ***: p < 0.001, ****: p < 0.0001 vs vehicle control or NAC-treated group (n = 3). [file 12935_2021_2150_MOESM3_ESM.docx]

**Figure S3.** Cinobufagin-induced oxidative DNA damage leads to replication stress and activation of DNA damage response. (**A**) Representative images of γH2AX immunostaining (scale bar: 10 μm) and quantification of γH2AX-positive cells. Cells were treated by 100 nM CBG for 3 h. At least 5000 cells per treatment group were analyzed. (**B-D**) Western blot. Cells were treated by 100 nM CBG for 3 h or the indicated times. n.s.: not significant, *: *p* < 0.05, **: *p* < 0.01, ***: *p* < 0.001, ****: *p* < 0.0001 vs vehicle control or NAC-treated group (n = 3).

**B**

**A**


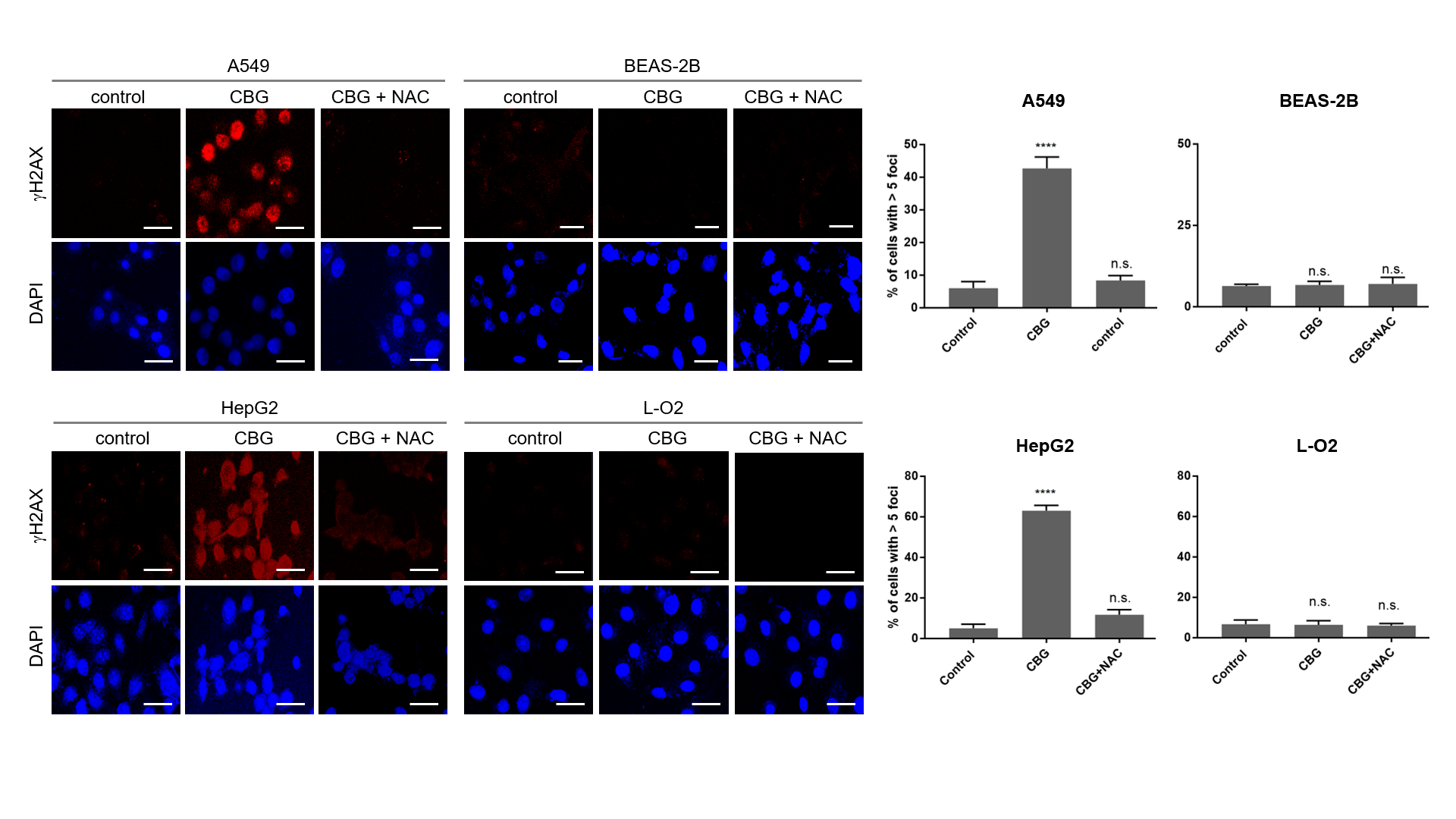

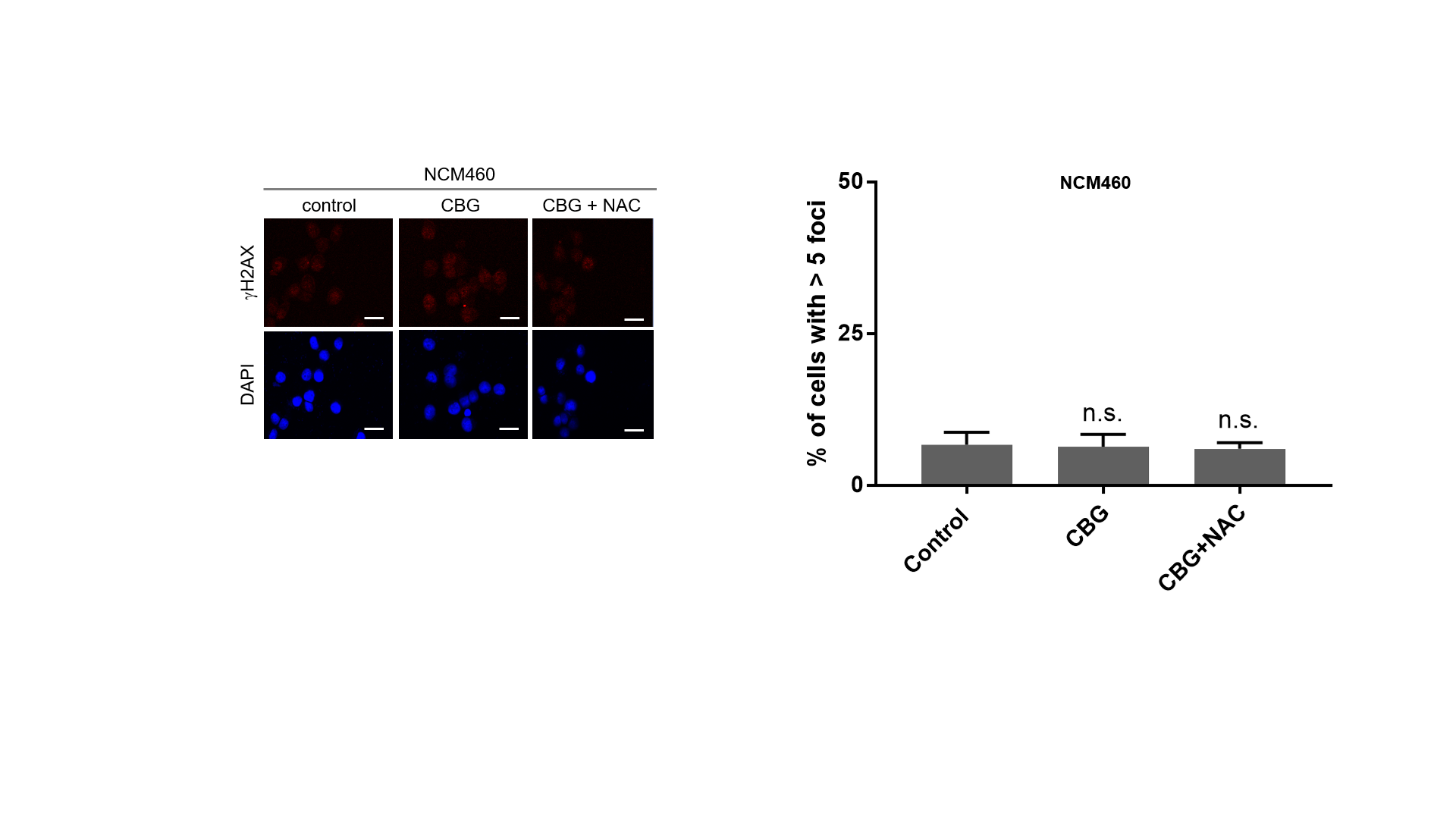

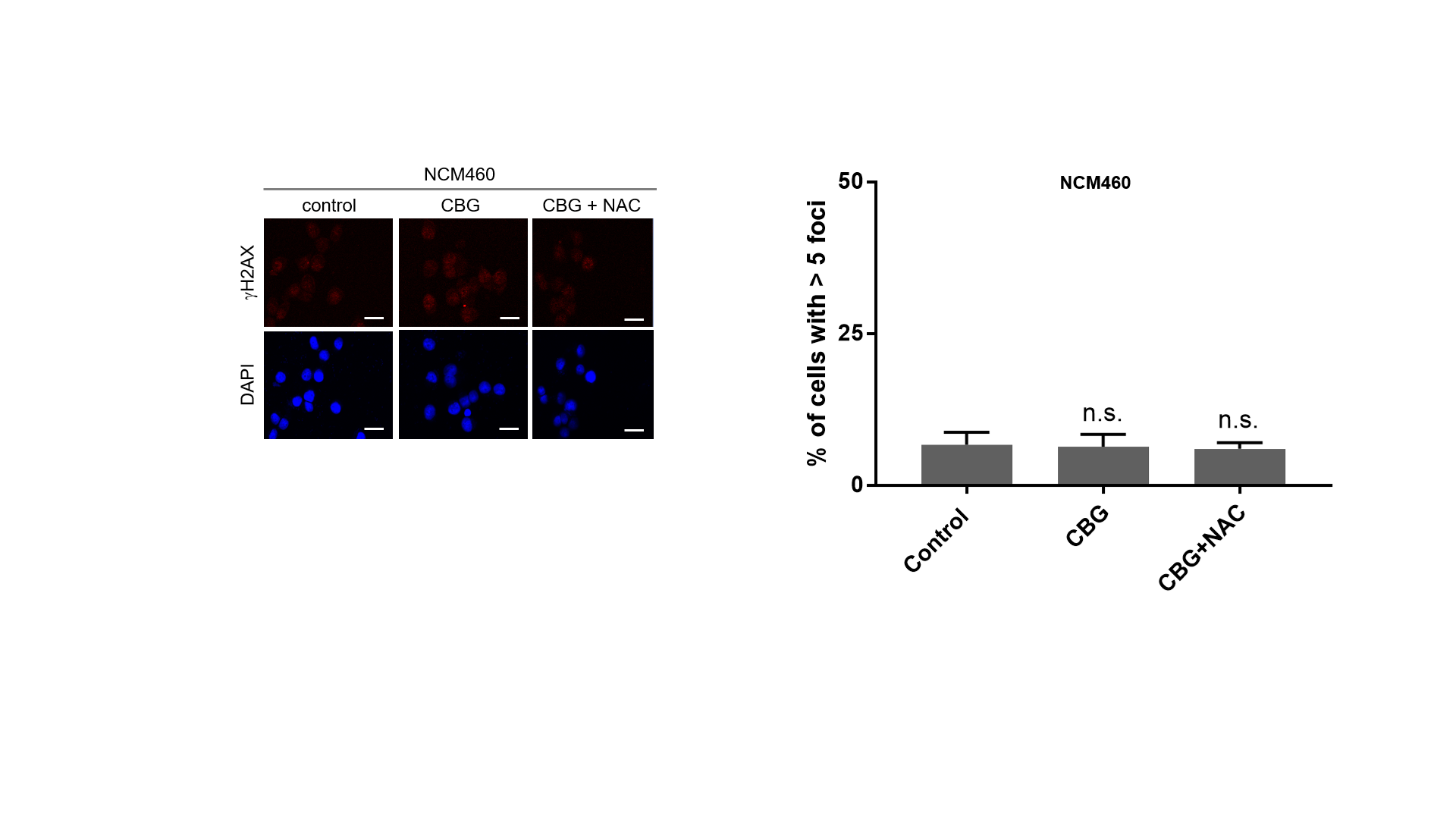


**NCM460**


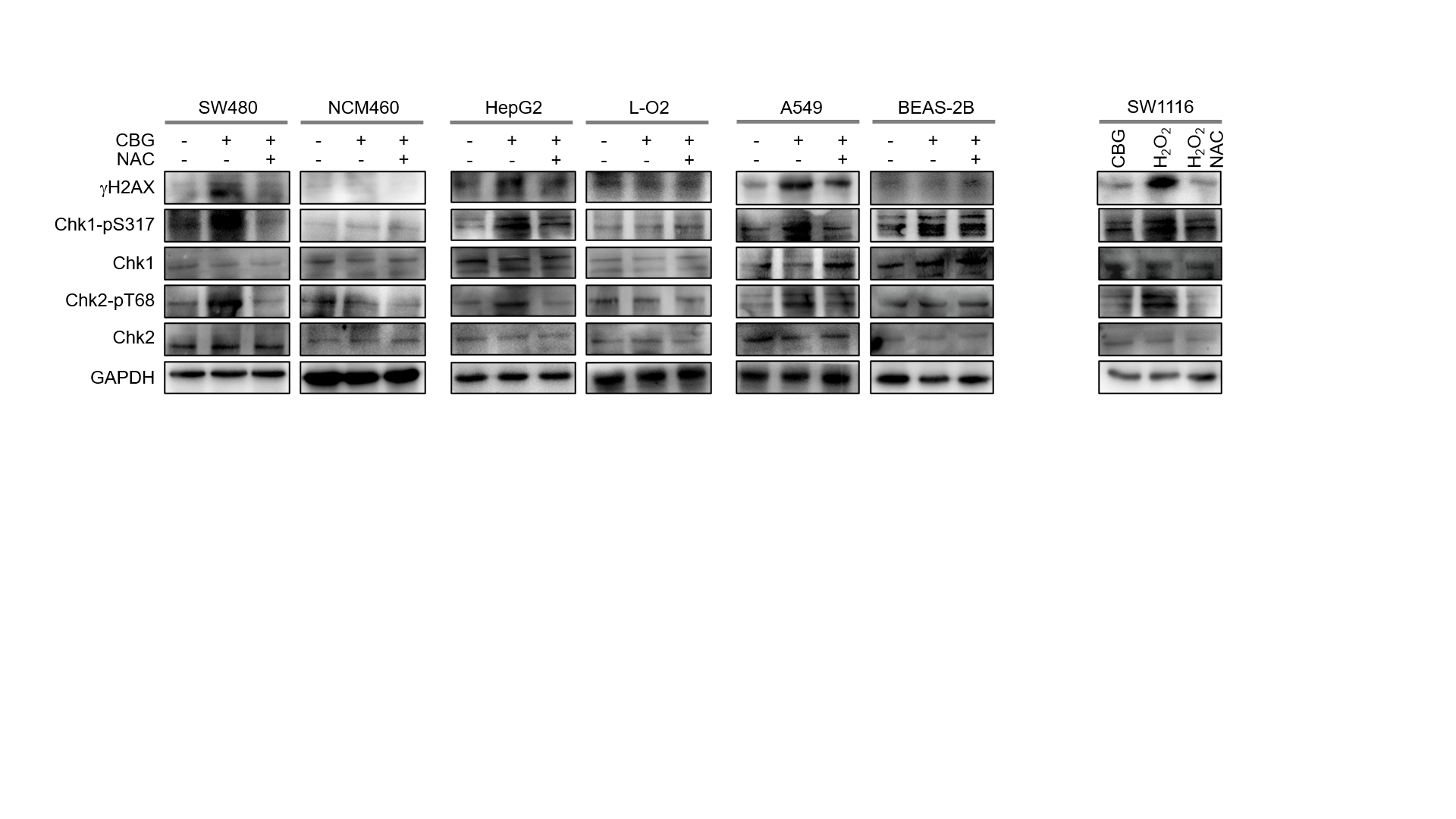

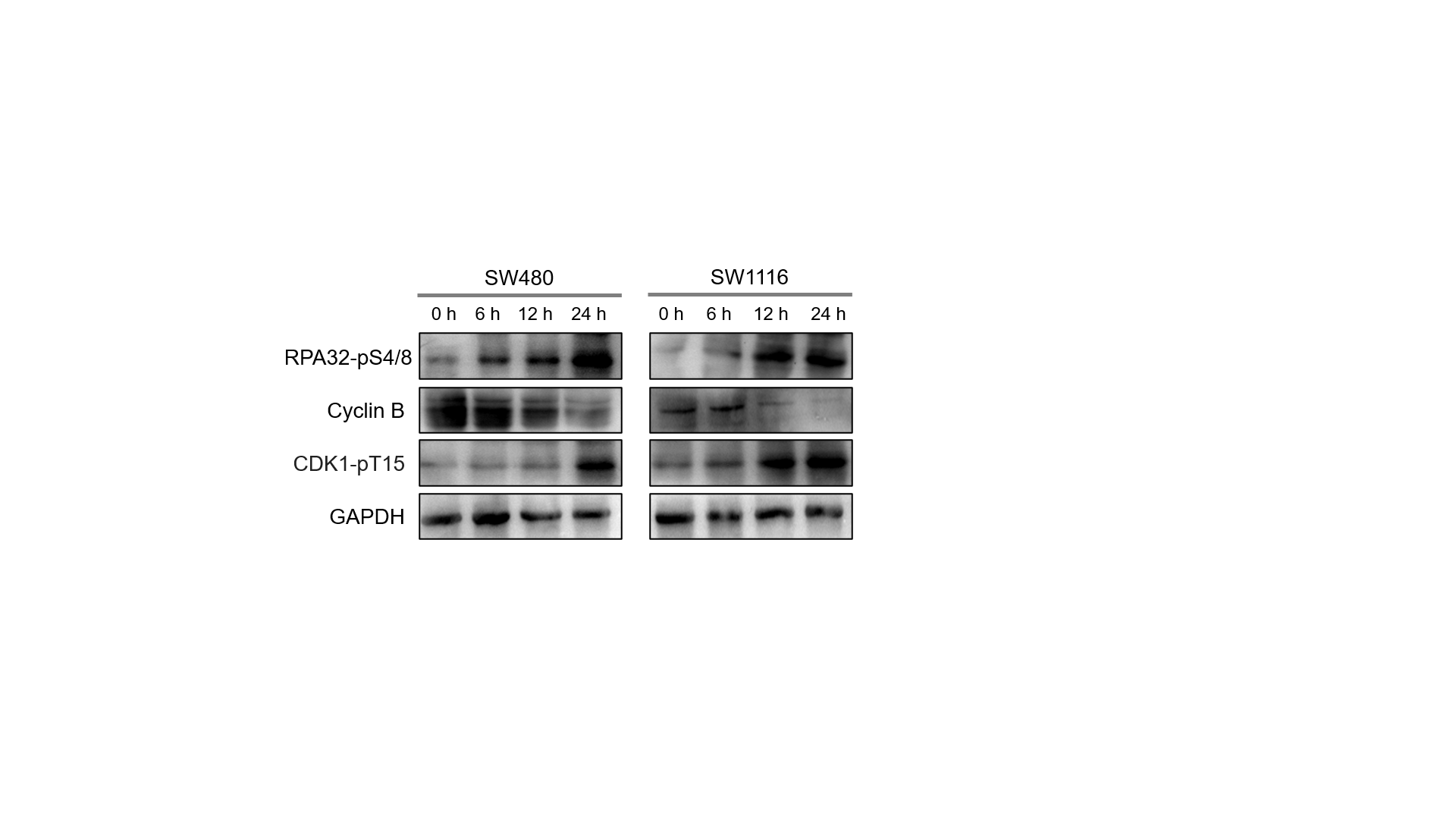


**C**


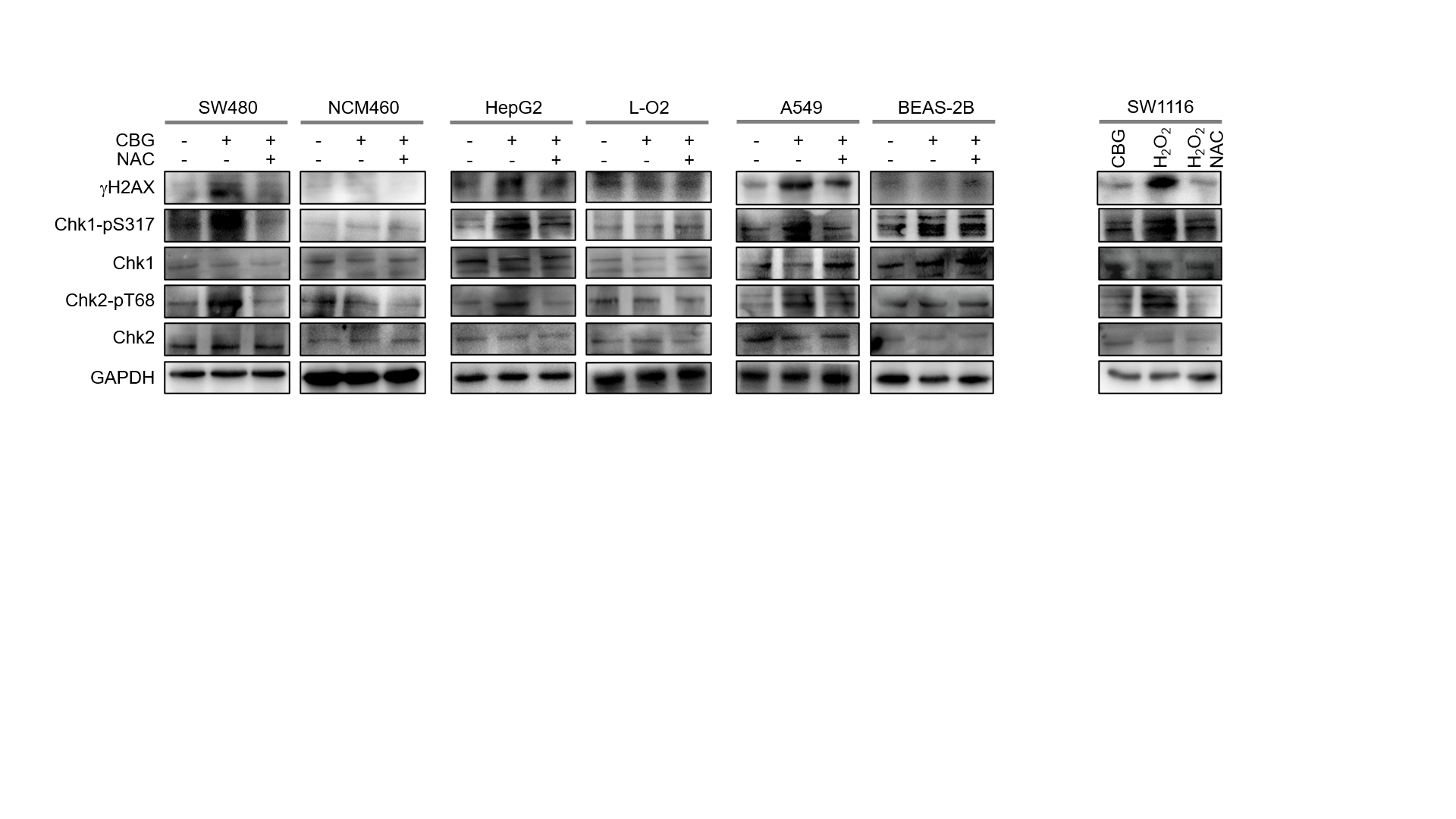

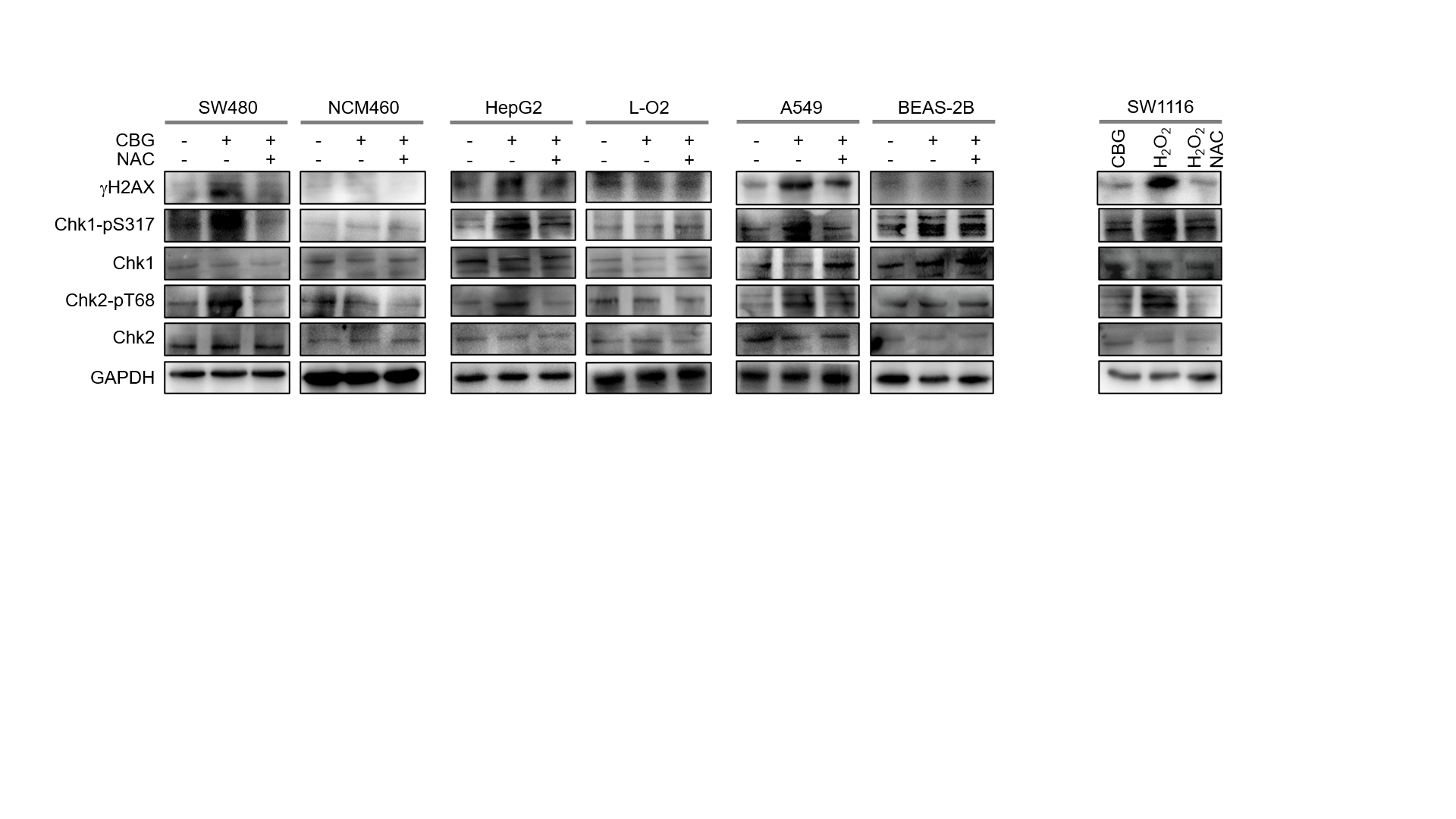


**D**
